# STING dependent BAX-IRF3 signaling results in apoptosis during late-stage *Coxiella burnetii* infection

**DOI:** 10.1038/s41419-024-06573-1

**Published:** 2024-03-08

**Authors:** Manish Chauhan, Chelsea A. Osbron, Heather S. Koehler, Alan G. Goodman

**Affiliations:** 1 School of Molecular Biosciences, College of Veterinary Medicine, Washington State University, Pullman, WA 99164 USA; 2 Paul G. Allen School for Global Health, College of Veterinary Medicine, Washington State University, Pullman, WA 99164 USA

**Keywords:** Apoptosis, Cell death and immune response

## Abstract

STING (STimulator of Interferon Genes) is a cytosolic sensor for cyclic dinucleotides (CDNs) and initiates an innate immune response upon binding to CDNs. *Coxiella burnetii* is a Gram-negative obligate intracellular bacterium and the causative agent of the zoonotic disease Q fever. The ability of *C. burnetii* to inhibit host cell death is a critical factor in disease development. Previous studies have shown that *C. burnetii* inhibits host cell apoptosis at early stages of infection. However, during the late-stages of infection, there is host cell lysis resulting in the release of bacteria to infect bystander cells. Thus, we investigated the role of STING during late-stages of *C. burnetii* infection and examined STING’s impact on host cell death. We show that the loss of STING results in higher bacterial loads and abrogates IFNβ and IL6 induction at 12 days post-infection. The absence of STING during *C. burnetii* infection significantly reduces apoptosis through decreased caspase-8 and -3 activation. During infection, STING activates IRF3 which interacts with BAX. BAX then translocates to the mitochondria, which is followed by mitochondrial membrane depolarization. This results in increased cytosolic mtDNA in a STING-dependent manner. The presence of increased cytosolic mtDNA results in greater cytosolic 2′-3′ cGAMP, creating a positive feedback loop and leading to further increases in STING activation and its downstream signaling. Taken together, we show that STING signaling is critical for BAX-IRF3-mediated mitochondria-induced apoptosis during late-stage *C. burnetii* infection.

## Introduction

The innate immune response serves as a primary defense mechanism for host cells during pathogenic microbial infection, mediated by phagocytic cells such as macrophages [1]. During this response, cellular pathogen recognition receptors (PRRs) engage in the recognition of molecular byproducts associated with invading pathogens [1, 2]. The detection of these pathogen-associated molecular patterns (PAMPs), such as pathogen-derived nucleic acids, by PRRs, triggers a signaling cascade leading to the activation of inflammatory and type I interferon (IFN) responses [3–5]. Stimulator of Interferon Genes (STING) is a transmembrane ER-resident protein that plays a key role in recognition of nucleic acids derived from invasive pathogens. STING functions downstream of the DNA sensor cyclic GMP-AMP synthase (cGAS), which detects cytosolic double-stranded DNA and metabolizes AMP and GMP into 2′,3′-cGAMP (cyclic[G (2′,5′)pA(3′,5′)p]) [6–8]. STING is then activated upon binding 2′,3′-cGAMP to initiate a signaling cascade inducing type I IFN [6]. In addition to cGAS-generated 2′,3′-cGAMP, STING can also bind directly to bacteria-generated cyclic dinucleotides (CDNs), such as cyclic dimeric guanosine monophosphate (c-di-GMP), cyclic dimeric adenosine monophosphate (c-di-AMP), and 3′,3′-cGAMP [9]. While STING was initially identified as a sensor for CDNs, it has multifaceted roles in innate immune responses during oncogenesis, autoinflammatory diseases, and microbial infections [10–14]. Previous studies have provided compelling evidence indicating that STING signaling plays a crucial role in activating apoptosis across a range of cell types. Notably, persistent activation of STING with agonists induces apoptosis in normal and malignant B cells [15]. Furthermore, in T cells, STING activation not only initiates an IFN response but also serves as a trigger for intrinsic apoptotic cell death [16]. In contrast, Gulen et al. revealed that murine macrophages do not undergo cell death when subjected to pharmacological activation of STING, due to their lower levels of STING expression in comparison to T cells, which have a robust STING response [17, 18]. These findings collectively underscore the importance of the intensity of STING activation in dictating whether the cellular outcome will be apoptosis or the induction of an interferon response. Gaidt et al. delved into this distinctive cell death program induced by STING activation in human monocytes, showing that STING signaling triggers lytic cell death with apoptosis-like features leading to NLRP3 activation [19]. Further investigation into the intricate mechanisms governing STING activation and its downstream consequences will undoubtedly shed more light on this intriguing interplay between cell death and immune responses.

*Coxiella burnetii*, a Gram-negative obligate intracellular bacterium, causes the zoonotic disease Q (query) fever. Infection with *C. burnetii* causes flu-like symptoms and may progress to severe chronic disease such as endocarditis and hepatitis [20, 21]. Additionally, due to its low infectious dose (1–10 bacteria), ability to be aerosolized, and its high environmental stability, *C. burnetii* is a potential bioterrorism agent [22]. The intracellular infection cycle of C. *burnetii* is complex and divided into the early stage (1–2 days), the replicative stage (4–6 days), and the late-stage (1–4 weeks) [23, 24]. As soon as it is phagocytized by macrophages, *C. burnetii* alters the membrane-bound compartment by subverting phagosome maturation within lysosome-derived organelles and begins multiplying within this *C. burnetii*-containing vacuole (CCV) [25]. During the early-stages of infection, *C. burnetii*’s infective small cell variant (SCV) differentiates into the large cell variant (LCV). The LCV becomes metabolically active and replicates, causing the expansion of the CCV [24, 26]. After rapidly multiplying, LCVs revert to infective SCVs during the late-stages of infection. SCVs are maintained inside the CCV for 2–4 weeks, followed by cell lysis and reinfection of bystander cells [27].

The phagolysosome-like niche of *C. burnetii* renders it immunologically silent, allowing *C. burnetii* replication within the CCV without activating PRRs [28, 29]. To subvert host innate mechanisms and maintain infection progression, *C. burnetii* utilizes the *Dot*/Icm type IVB secretion system (T4BSS) [30]. *C. burnetii* manipulates host processes by translocating bacterial effector proteins through its T4BSS to the host cytosol. *C. burnetii* effector proteins target host mediated apoptosis, membrane trafficking pathways, among other host cell pathways [29, 31–34]. The inhibition of host cell intrinsic and extrinsic apoptosis pathways plays a crucial role in disease progression during *C. burnetii* infection [35, 36]. Previously, it was shown that *C. burnetii* infection inhibits cytochrome c release from the mitochondria, preventing caspase-3 activation [32]. Similarly, human THP-1 cells and primary alveolar macrophages from cynomolgus macaques treated with the apoptotic inducer staurosporine exhibited reduced caspase-9, caspase-3, and poly (ADP-ribose) polymerase (PARP) cleavage [37]. Inhibition of bacterial RNA or protein synthesis by rifampin or chloramphenicol reduces the apoptotic arrest caused by *C. burnetii* on staurosporine-treated infected cells [37]. The *C. burnetii* effector protein AnkG (ankyrin repeat (Ank) family) translocates to the cytosol via the T4BSS and inhibits the host apoptosis pathway [38].

Previous studies investigated how *C. burnetii* inhibits apoptosis during the early stages of infection [27, 37–40]. While a study conducted by Bradly et al. has suggested that STING does not appear to play a significant role during the initial stages of *C. burnetii* infection in mouse macrophages [41], further investigation is required to understand host response pathways that are activated during late-stages of *C. burnetii* infection and how these processes influence manifestation of chronic infection leading to serious clinical conditions. Better understanding of host factors during late-stage infection will help to reduce the relapse of Q fever and shorten the interval of therapeutic regime, which typically lasts several years [21, 20]. In this study, we investigated the role of STING during *C. burnetii* infection and its impact on host cell death. We found that the absence of STING in mouse bone marrow-derived macrophages (BMDMs) allows higher bacterial replication and reduced cell death during infection by late times post-infection. We observed diminished caspase-8 and caspase-3 activation that resulted in reduced apoptosis when STING was absent. During *C*. *burnetii* infection, STING activation is required for BAX-IRF3 interaction and the induction of BAX-mediated mitochondrial depolarization. STING-dependent mtDNA release following mitochondrial depolarization resulted in increased 2′,3′-cGAMP levels, triggering a positive feedback loop to further STING activation. Altogether, we demonstrate that STING signaling is critical for BAX-IRF3-mediated mitochondria-induced apoptosis during late-stages of *C. burnetii* infection.

## Results

### STING deficiency leads to increased bacterial load in BMDMs and L929 cells

**Fig. 1:**
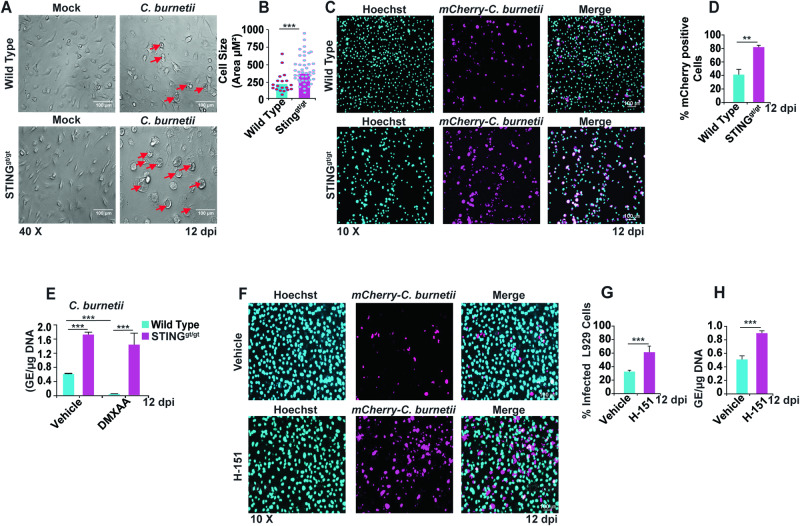
STING deficiency leads to increased bacterial load. **A** Differential interference contrast (DIC) micrograph of mock- and *C. burnetii*-infected WT and STING^gt/gt^ BMDMs at 12 dpi. Red arrows indicate rounded, enlarged cells. **B** Graph represents the quantified cell size of *C. burnetii* infected WT and STING^gt/gt^ BMDMs at 12 dpi. Data are representative of three different fields of view from three biological replicates of each genotype from three independent experiments. Error bars, SEM. Unpaired *T*-test, ***p < 0.001. **C** Fluorescent micrographs show mCherry-*C. burnetii* infected (magenta) WT and STING^gt/gt^ BMDMs and counterstained with nuclear staining dye Hoechst 33342 (cyan). The micrographs are representative of four independent experiments. **D** Percentage of mCherry-positive WT and STING^gt/gt^ BMDMs at 12 dpi. Data are representative of three different fields of view from three biological replicates of each genotype from three independent experiments. Error bars, SEM. Unpaired *T*-test, **p < 0.01. **E** Bacterial load in WT and STING^gt/gt^ BMDMs at 12 dpi measured as genomic equivalents (GE) in presence and absence of pharmacological STING activator DMXAA (25 μg/ml). Data are representative of three biological replicates of 2 × 10^5^ cells per well of each genotype from three independent experiments. Error bars, SEM. Unpaired *T*-test, ***p < 0.001. **F** Fluorescent micrographs show mCherry-*C. burnetii* infected (magenta) L929 cells treated with vehicle or H-151 and counterstained with Hoechst 33342 (cyan) at 12 dpi. The micrographs are representative of five independent experiments. **G** Percentage of mCherry-positive vehicle- or H-151(2 μM)-treated L929 cells at 12 dpi. Data are representative of five different fields of view from three wells from each treatment group from three independent experiments. Error bars, SEM. Unpaired *T*-test, ***p < 0.001. **H** Bacterial load in vehicle- or H-151-treated L929 cells at 12 dpi measured as genomic equivalents (GE). Data are representative of five biological replicates from 5 × 10^5^ cells per well of each genotype from three independent experiments. Error bars, SEM. Unpaired *T*-test, ***p < 0.001.

To determine the effect of STING on *C. burnetii* infection, wild-type (WT) and STING-deficient (STING^gt/gt^) mouse bone marrow-derived macrophages (BMDMs) were mock-infected or infected with *C. burnetii* at a multiplicity of infection (MOI) of 100 genome equivalents (GE)/cell. Morphological changes such as rounding up and enlargement of *C. burnetii*-infected cells were observed after 12 days post-infection (dpi). Interestingly, infected STING^gt/gt^ BMDMs exhibited more rounded and enlarged cells than WT BMDMs, as indicated with red arrows (Fig. 1A). Quantification of *C. burnetii*-infected BMDMs shows that STING^gt/gt^ BMDMs were larger in size compared to WT BMDMs (Fig. 1B). The morphological changes associated with *C. burnetii* infection were not as prominent at earlier dpi (Fig. S1A). To link these morphological changes in cell shape with the intracellular bacterial load, WT and STING^gt/gt^ BMDMs were then infected with mCherry expressing *C. burnetii* (mCherry-*C. burnetii)* at a MOI of 100 GE/cell to determine the percent infectivity (Fig. 1C). At 12 dpi, infected STING^gt/gt^ BMDMs exhibit more mCherry-positive cells than infected WT BMDMs (Fig. 1C, D).

To corroborate the role of STING we found using our BMDM genetic model, we next tested the effect of STING on *C. burnetii* infection using pharmacologic activation and inhibition. First, we measured bacterial replication during *C. burnetii* infection in BMDMs by quantifying genome equivalents in the presence and absence of the STING agonist, DMXAA. These results demonstrate that the prior activation of STING inhibits bacterial replication (Figs. 1E and S1B).

Next, L929 mouse fibroblasts were treated with the STING inhibitor H-151 [42] and infected with mCherry-*C. burnetii* at a MOI of 100 GE/cell. Inhibition of STING with H-151 increased mCherry-positive L929 cells at 12 dpi (Fig. 1F, G). Similarly, GE quantification of STING-inhibited L929 cells shows increased bacterial replication at 12 dpi (Fig. 1H). Taken together, these results suggest that STING deficiency permits increased bacterial replication in primary mouse macrophages and a fibroblast cell line.

### STING is critical for a cytokine response during *C. burnetii* infection

**Fig. 2:**
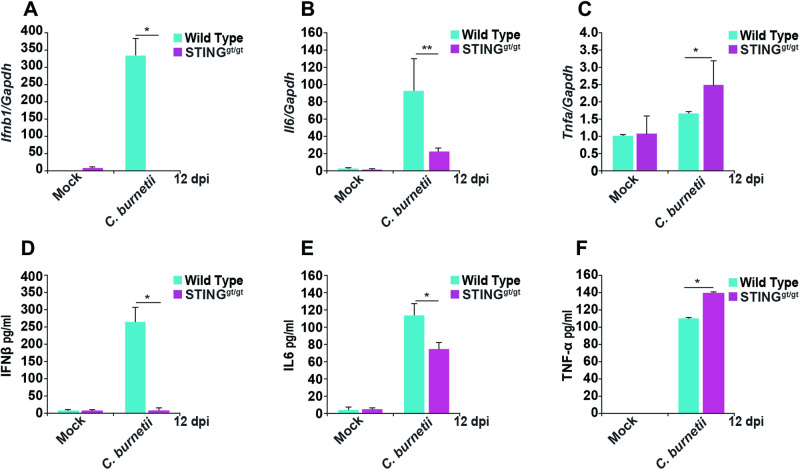
Host response to *C. burnetii* infection in the presence and absence of STING. **A**–**C** qRT-PCR of *Ifnb, Il6*, and *Tnfa* in mock- or *C. burnetii*-infected WT and STING^gt/gt^ BMDMs at 12 dpi. Data are representative of four biological replicates from 2 × 10^5^ cells per well of each genotype from four independent experiments. Error bars, SEM. Unpaired *T*-test, *p < 0.05, **p < 0.01. **D**–**F** ELISA of IFNβ, IL6 and TNFα produced in cell culture supernatants of mock- or *C. burnetii*-infected WT and STING^gt/gt^ BMDMs at 12 dpi. Data are representative of three biological replicates each from 2 × 10^5^ cells per well of each genotype from three independent experiments. Error bars, SEM. Unpaired *T*-test, *p < 0.05.

STING-induced type I IFN responses play a key role in innate immune responses against DNA viruses [43]. To investigate whether *C. burnetii* infection in BMDMs induces a type I IFN response in a STING-dependent manner, we measured gene expression and cytokine production. At the transcript level, infected WT BMDMs exhibited robust *Ifnb1, Il-1 receptor antagonist (Il-1ra)*, and *Il6* induction, whereas these cytokines exhibit reduced induction in the absence of STING at 12 dpi (Figs. 2A, B and S2A). However, at early stages of *C. burnetii* infection (1 and 3 dpi), induction of *Ifnb1* and *Il6* were similar in WT and STING^gt/gt^ BMDMs (Fig. S2B–E). Interestingly, we observed increased *Tnfα* expression in the absence of STING (Fig. 2C). We also detected decreased IFNβ and IL6 levels in the cell culture supernatant of STING^gt/gt^ BMDMs (Fig. 2D, E). Consistent with the transcript level of *Tnfα*, infected STING^gt/gt^ BMDMs secreted higher amounts of TNFα than WT BMDMs (Fig. 2F). Collectively, these results suggest that infection with *C. burnetii* triggers a STING-dependent cytokine responses by 12 dpi in mouse BMDMs.

### *C. burnetii* induces apoptotic cell death in mouse BMDMs in a STING-dependent manner

**Fig. 3:**
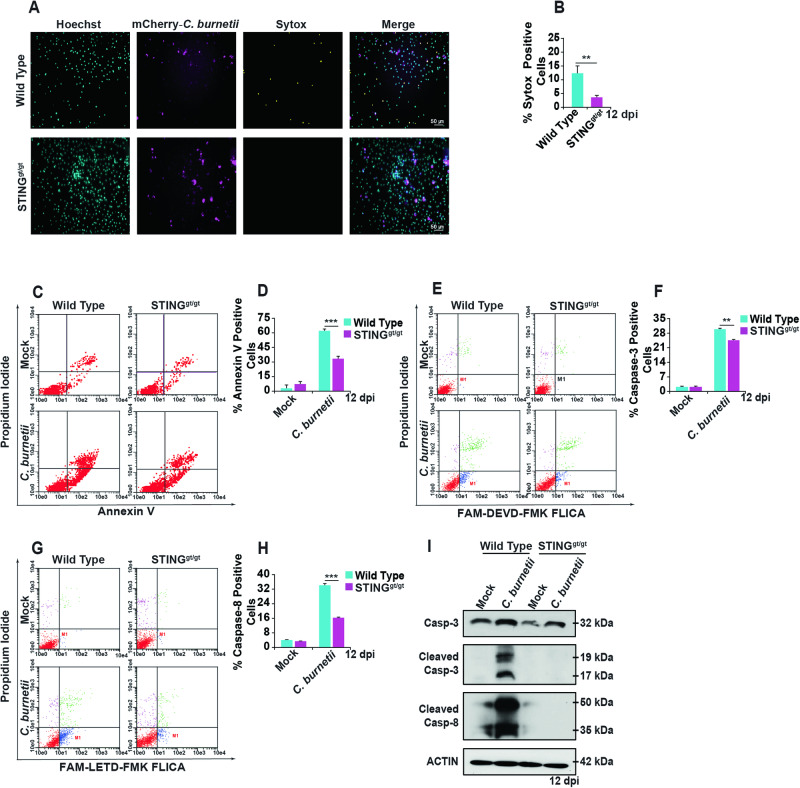
STING promotes programmed cell death during *C. burnetii* infection in BMDMs. **A** Fluorescent micrographs of mCherry-*C. burnetii*-infected (magenta) WT and STING^gt/gt^ BMDMs at 12 dpi, Hoechst 33342 (cyan) nuclear staining, and SYTOX (yellow) staining to detect dead cells. The micrographs are representative of three independent experiments. **B** Percentage of SYTOX-positive WT and STING^gt/gt^ BMDMs during *C. burnetii* infection at 12 dpi. Data are representative of three different fields of view from four independent experiments. Error bars, SEM. Unpaired *T*-test, **p < 0.01. **C** Annexin V- and Propidium Iodide-positive cell populations from mock- and *C. burnetii*-infected WT and STING^gt/gt^ BMDMs at 12 dpi using flow cytometry. Representative scatter plots from three independent experiments are shown. **D** Percentage of Annexin V-positive WT and STING^gt/gt^ BMDMs population during *C. burnetii* infection at 12 dpi. Data are representative of three biological replicates from three independent experiments. Error bars, SEM. Unpaired *T*-test, ***p < 0.001. **E** Caspase-3 activation was determined by flow cytometry using the FAM-DEVD-FMK FLICA peptide in mock- or *C. burnetii*-infected WT and STING^gt/gt^ BMDMs at 12 dpi. Representative scatter plots from three independent experiments are shown. **F** Percentage of caspase-3 positive WT and STING^gt/gt^ BMDMs during *C. burnetii* infection at 12 dpi. Data are representative of three biological replicates from three independent experiments. Error bars, SEM. Unpaired *T*-test, **p < 0.01. **G** Caspase-8 activation was determined by flow cytometry using the FAM-LETD-FMK FLICA peptide in mock- or *C. burnetii*-infected WT and STING^gt/gt^ BMDMs at 12 dpi. Representative scatter plots from three independent experiments are shown. **H** Percentage of caspase-8 positive WT and STING^gt/gt^ BMDMs during *C. burnetii* infection at 12 dpi. Data are representative of three biological replicates from three independent experiments. Error bars, SEM. Unpaired *T*-test, ***p < 0.001. **I** Caspase-3 and caspase-8 activation in mock- or *C. burnetii*-infected WT and STING^gt/gt^ BMDMs was determined by western blot at 12 dpi. Blots are representative of three independent biological replicates.

Previous studies suggest that STING activation induces IFN production, leading to cell death in T cells [16]. Therefore, we assessed cell death using a live cell impermeant nucleic acid stain, SYTOX Green, during *C. burnetii* infection in WT and STING^gt/gt^ BMDMs. At 12 dpi, there were fewer SYTOX-positive cells in infected STING^gt/gt^ BMDMs than in WT BMDMs (Figs. 3A, B and S3A). Whereas sterile activation of STING with pharmacological activator DMXAA does not induce cell death in WT and STING^gt/gt^ BMDMs (Fig. S3A). These data indicate that STING activation induces cell death during *C. burnetii* infection. In the absence of STING, there is increased host cell survival, despite STING^gt/gt^ BMDMs having higher bacterial load than WT BMDMs.

Apoptotic cell death is a crucial host defense mechanism against invading pathogens [44]. To survive intracellularly at the early stages of infection, *C. burnetii* inhibits host cell apoptosis [27, 37–40]. Previous studies have primarily examined the early stages of infection (1–3 dpi). Therefore, we hypothesized that the cell death observed during the later stages of infection may be apoptotic in nature, due to the role that STING plays in apoptosis [45]. Analysis of cell death at 12 dpi by flow cytometry demonstrated that the absence of STING in *C. burnetii*-infected BMDMs significantly reduces apoptosis (Fig. 3C, D). At 1, 3, and 6 dpi, there was no significant difference in apoptosis in mock or infected WT and STING^gt/gt^ BMDMs (Fig. S3C). However, further analysis of apoptotic signaling during later stages of *C. burnetii* infection revealed significant differences in the activation of caspase-3 and caspase-8 between infected STING^gt/gt^ BMDMs and WT BMDMs. Specifically, our results indicate that infected STING^gt/gt^ BMDMs have lower caspase-3 (Fig. 3E, F) and caspase-8 (Fig. 3G, H) activation compared to infected WT BMDMs at 12 dpi. Furthermore, western blot analysis validated our flow cytometry results, demonstrating that infected STING^gt/gt^ BMDMs exhibit decreased cleaved caspase-3 and caspase-8 than WT BMDMs (Fig. 3I). Based on these data, it is evident that STING plays an essential role in apoptosis during the late-stages of *C. burnetii* infection.

### STING induces mitochondria-mediated apoptosis through a BAX/IRF3 signaling during *C. burnetii* infection

**Fig. 4:**
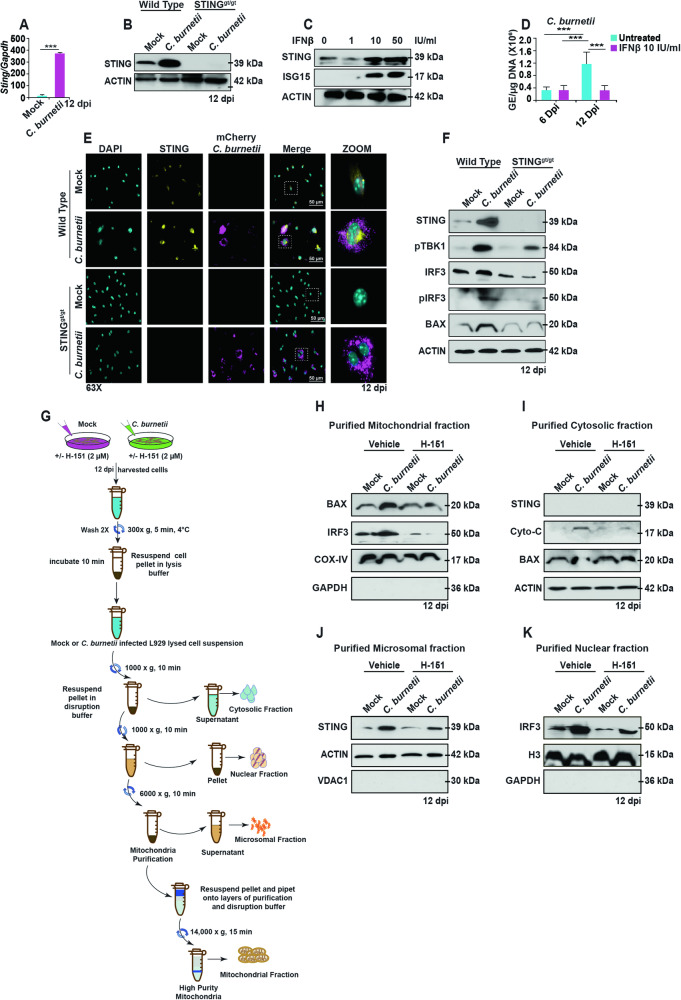
Elevated STING levels during *C*. *burnetii* infection induce mitochondria-mediated apoptosis through the BAX-IRF3 pathway. **A** qPCR of *Tmem173* (*Sting*) from mock- or *C. burnetii*-infected WT BMDMs at 12 dpi. Data are representative of four biological replicates each of 5 × 10^5^ cells per well of each genotype from three independent experiments. Error bars, SEM. Unpaired *T*-test, ***p < 0.001. **B** STING protein levels in mock- or *C. burnetii*-infected WT and STING^gt/gt^ BMDMs was determined by western blot at 12 dpi. **C** Western blot analysis of WT BMDMs treated with IFNβ to probe STING and ISG15 levels. **D** Bacterial load in WT BMDMs measured as genomic equivalents (GE) in presence and absence of IFNβ (10 IU/ml). Data are representative of three biological replicates of 2 × 10^5^ cells per well of each genotype from three independent experiments. Error bars, SEM. Unpaired *T*-test, ***p < 0.001. **E** Fluorescent micrographs showing localization of STING (yellow) in mock- or mCherry-*C. burnetii* (magenta)-infected WT and STING^gt/gt^ BMDMs, counterstained with DAPI (cyan), at 12 dpi. The micrographs are representative of at least three independent experiments. **F** Western blot analysis of STING signaling components in mock- or *C. burnetii*-infected WT and STING^gt/gt^ BMDMs using the indicated antibodies at 12 dpi. **G** Schematic for cell fractionation for mock- and *C. burnetii-*infected L929 cells at 12 dpi treated with vehicle or H-151. **H** Western blot analysis of BAX, IRF3, and COX-IV from purified mitochondrial fractions. **I** Western blot analysis of STING, cytochrome c, and BAX from purified cytosolic fractions. **J** Western blot analysis of STING in purified microsomal fractions. **K** Western blot analysis of IRF3 and Histone H3 in purified nuclear fractions. All blots are representative of three independent experiments.

According to Gulen et al., the strength of STING signaling and its intracellular levels may affect its apoptotic function [17]. To investigate this, we assessed STING mRNA and protein levels during late-stages of *C. burnetii* infection. Our results indicate that at 12 dpi, both STING mRNA and protein levels in *C. burnetii*-infected WT BMDMs were significantly higher compared to mock-infected cells (Fig. 4A, B). Furthermore, we examined STING levels throughout infection (1, 3, 6, 12 dpi) and observed the greatest increase in STING expression at 12 dpi (Fig. S4A). We also investigated the impact of different *C. burnetii* MOIs on STING levels at 12 dpi and observed the greatest STING expression at an MOI of 100 GE/cell (Fig. S4B). These results suggest a time- and dose-dependence of STING levels during *C. burnetii* infection.

Previous research has established that the expression of STING can be triggered by type I IFN (e.g., IFNβ), thus setting up a positive feedback loop that amplifies its induction. This mechanism suggests a self-reinforcing cycle wherein the presence of type I IFN leads to increased STING expression, potentially strengthening the cellular response to pathogenic stimuli [46]. To demonstrate the impact of IFNβ on STING protein levels, we treated WT BMDMs with increasing concentration of mouse IFNβ. Our observations revealed a dose-dependent induction of both STING and ISG15 (IFN-stimulated gene 15) levels (Fig. 4C). Additionally, we tested the effects of IFNβ on *C. burnetii* replication in WT BMDMs and observed that IFNβ-treated cells exhibited reduced bacterial replication at 12 dpi (Fig. 4D). These findings exhibit the inhibitory effect of IFNβ on bacterial proliferation within infected cells.

In its inactive state, STING is an ER-resident protein with dispersed localization throughout ER. Upon activation, STING translocates to the Golgi/ER-Golgi intermediate compartment (ERGIC), leading to TBK1 and IRF3 phosphorylation [43, 47–49]. Therefore, we examined STING subcellular localization during *C. burnetii* infection in WT and STING^gt/gt^ BMDMs. Notably, we observed higher levels of STING in *C. burnetii*-infected WT BMDMs compared to mock-infection, and the localization of STING appeared more concentrated than dispersed, indicating the activation of STING during *C. burnetii* infection (Fig. 4E). We also verified the translocation of STING to the ERGIC following activation using DMXAA, and we observed STING translocation to ERGIC compartments following DMXAA treatment (Fig. S4C). This demonstrates that sterile activation can mimic the translocation process observed during pathogenic activation.

We next investigated the activation of downstream STING signaling components, specifically TBK1 and IRF3 phosphorylation. Our results show that during *C. burnetii* infection, infected WT BMDMs exhibited increased TBK1 and IRF3 phosphorylation, whereas the absence of STING reduced the phosphorylation of TBK1 and IRF3 (Fig. 4F). The presence of residual phosphorylated TBK1 observed in *C. burnetii*-infected STING^gt/gt^ BMDMs might potentially be attributed to TLR (Toll-like receptor) activation [41]. IRF3 activation has been demonstrated to trigger BAX-mediated apoptosis by binding with BAX in the cytosol and translocating to the mitochondrial membrane [50–53]. To investigate the role of STING in IRF3-induced BAX-mediated apoptosis, we examined the levels of BAX through western blot analysis. Our results show lower levels of BAX in the absence of STING, whereas infected WT BMDMs exhibited higher levels of BAX at 12 dpi, suggesting a possible role for STING in IRF3-induced BAX-mediated apoptosis (Fig. 4F). Additionally, similar results were observed using H-151 inhibition of STING in L929 cells infected with *C. burnetii* at 12 dpi (Fig. S4D).

To investigate whether STING-mediated apoptosis is attributed to BAX activation, L929 cells were infected with *C. burnetii* and treated with H-151, followed by fractionation of cells to isolate purified mitochondria, cytosol, microsomes, and nuclei at 12 dpi (Fig. 4G). The purified cellular fractions were analyzed by western blot to detect IRF3 and BAX in the mitochondria. The results indicate increased levels of BAX and IRF3 in the purified mitochondrial fraction of infected L929 cells, whereas their levels were lower in the H-151-treated cells (Fig. 4H). In the cytosolic fraction, there was increased cytochrome c in the vehicle-treated *C. burnetii-*infected L929 cytosolic fraction compared to H-151-treated cells (Fig. 4I). In the microsomal and nuclear fractions, higher levels of STING and IRF3, respectively, were detected in *C. burnetii-*infected L929 cells compared to mock-infected L929 cells (Fig. 4J, K). The reduced levels of IRF3 and BAX in the mitochondrial fraction of H-151-treated *C. burnetii*-infected L929 cells strengthens our observation that STING activation is necessary for the translocation of BAX/IRF3 to mitochondria and the release of cytochrome c to the cytosol.

### IRF3 interaction with BAX induces its translocation to mitochondria in a STING-dependent manner

**Fig. 5:**
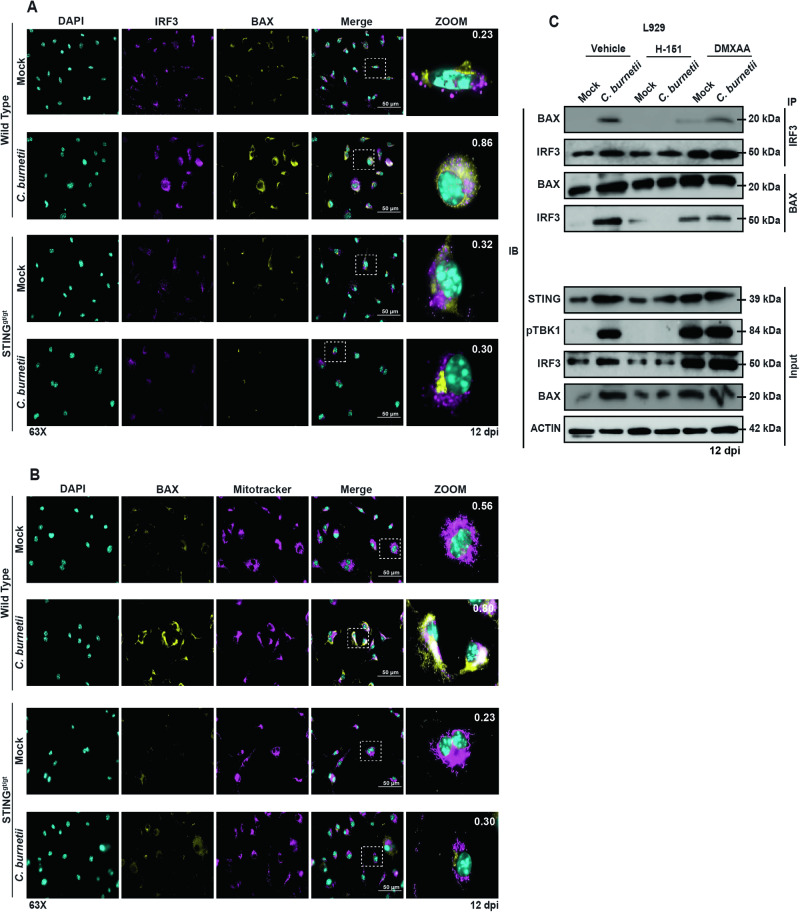
Subcellular localization of STING and it’s signaling partners during *C. burnetii* infection. **A** Micrographs show colocalization of IRF3 (magenta) and BAX (yellow) in mock- or *C. burnetii*-infected WT and STING^gt/gt^ BMDMs. The micrographs are representative of at least three independent experiments. Pearson’s correlation coefficient for localization overlap is shown on the zoomed micrograph. **B** The micrographs show colocalization of BAX (yellow) and Mitotracker (magenta) mitochondria staining in mock- or *C. burnetii*-infected WT and STINGgt/gt BMDMs. The micrographs are representative of at least three independent experiments. Pearson’s correlation coefficient for localization overlap is shown on the zoomed micrograph. **C** Western blot analysis using the indicated antibodies following immunoprecipitation of IRF3 from mock- or *C. burnetii*-infected L929 cells treated with vehicle or H-151 or DMXAA. All blots are representative of three independent experiments.

Next, we investigated the subcellular localization of BAX and IRF3 during *C. burnetii* infection in WT and STING^gt/gt^ BMDMs at 12 dpi. Fluorescence microscopy images show BAX and IRF3 colocalization in *C. burnetii*-infected WT BMDMs, as suggested by a high Pearson’s correlation coefficient (PCC) of 0.86, whereas the colocalization is absent in STING^gt/gt^ BMDMs (PCC = 0.30) (Fig. 5A). We also examined the colocalization of BAX with IRF3 during DMXAA-induced STING activation. Both DMXAA and *C. burnetii* infection led to strong colocalization of BAX and IRF3 (PCC range 0.83–0.90) (Fig. S5A). To confirm BAX translocation to the mitochondria during *C. burnetii* infection of BMDMs, cells were stained with CMXROS Mitotracker, followed by staining for BAX. Our results indicate that BAX colocalized with the mitochondria in infected WT BMDMs (PCC = 0.80), whereas the colocalization was lost in infected STING^gt/gt^ BMDMs (PCC = 0.30) (Fig. 5B).

The interaction of BAX and IRF3 was also tested by co-immunoprecipitation. BAX and IRF3 co-immunoprecipitated in vehicle-treated, *C. burnetii*-infected or DMXAA-treated L929 cells, but in the presence of the STING inhibitor H-151, the interaction between IRF3 and BAX was absent. However, activation of STING with the DMXAA agonist induces BAX interaction with IRF3 (Fig. 5C). Prior research indicates a relationship between the induction of STING levels and increases in BAX expression [54]. Based on our results, interaction between IRF3 and BAX in the cytosol is dependent on the presence of STING. This interaction activates BAX, which then translocates to the mitochondria to induce membrane, causing cytochrome c leakage to the cytosol and the induction of apoptosis.

### STING activation during *C. burnetii* infection induces mitochondrial depolarization leading to increased ROS and calcium levels in mouse BMDMs

**Fig. 6:**
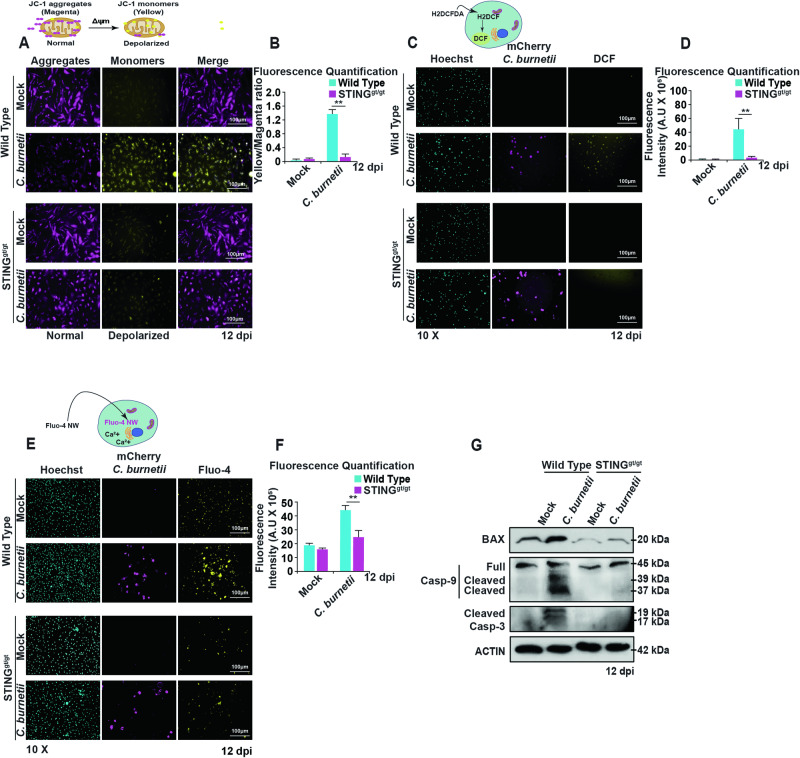
STING-dependent mitochondrial damage during *C. burnetii* infection. **A** Fluorescence micrographs exhibiting mitochondrial potential of mock- and *C. burnetii*-infected WT and STING^gt/gt^ BMDMs using JC-1 dual fluorescence mitochondrial dye at 12 dpi. Mitochondria with normal polarization show dye aggregate (magenta) and depolarized mitochondria show dye monomer (yellow). The micrographs are representative of three independent experiments. **B** Quantitation of fluorescence in (**A**) using ratios of yellow (low membrane potential) to magenta (high membrane potential). Depolarization of mitochondria is shown by the increase in the ratio of yellow to magenta. The micrographs are representative of three independent experiments. **C** Fluorescence micrographs of H2DCFDA (yellow) for reactive oxygen species (ROS) of mock- and mCherry-*C. burnetii* (magenta)-infected WT and STING^gt/gt^ BMDMs at 12 dpi. The micrographs are representative of three independent experiments. **D** Quantitation of fluorescence in (**C**). **E** Fluorescence micrographs of Fluo-4 NW (magenta) for calcium ion detection mock- and mCherry-*C. burnetii*-infected WT and STING^gt/gt^ BMDMs at 12 dpi. **F** Quantitation of fluorescence in (**E**). **G** Western blot showing mitochondria-mediated apoptosis pathway activation in mock- and *C. burnetii*-infected WT and STING^gt/gt^ BMDMs using the indicated antibodies. Blots are representative of at least three independent experiments. In (**B**), (**D**) and (**F**) data are representative of three biological replicates from two independent experiments. Error bars, SEM. Unpaired *T*-test, **p < 0.01.

It has been demonstrated that BAX translocation to the mitochondria in response to virus infection or alcohol uptake induces mitochondria-mediated apoptosis [50, 55]. Building on this premise, we aimed to examine how STING-mediated BAX localization to the mitochondria impacts mitochondrial membrane potential. We examined mitochondrial transmembrane potential using the JC-1 assay (ΔΨm) on mock- or *C. burnetii-*infected WT and STING^gt/gt^ BMDMs. The results indicate that at 12 dpi, mock-infected WT and STING^gt/gt^ BMDMs have functional mitochondria displaying higher levels of JC-1 aggregates (shown in magenta) than JC-1 monomers (shown in yellow) (Fig. 6A, B). Similar levels of JC-1 aggregates were observed in *C. burnetii-*infected STING^gt/gt^ BMDMs at 12 dpi. In contrast, *C. burnetii-*infected WT BMDMs displayed mitochondria with elevated levels of JC-1 monomers, indicating depolarized mitochondria with reduced membrane potential (Fig. 6A, B).

Our results so far suggest that STING reduces mitochondrial health in the context of *C. burnetii* infection. Prior studies have indicated that exposure to anticancer drugs result in mitochondrial depolarization upon treatment, which subsequently triggers the generation of reactive oxygen species (ROS) and eventually leads to cell death [56–59]. Based on the results of our JC-1 mitochondrial transmembrane potential assay, we speculated that mitochondrial depolarization of WT BMDMs could result in increased ROS production during infection. To test this hypothesis, we examined ROS levels in *C. burnetii-*infected WT and STING^gt/gt^ BMDMs using a ROS indicator dye. Our results indicate that ROS levels are lower in *C. burnetii*-infected STING^gt/gt^ BMDMs compared to WT BMDMs (Fig. 6C, D).

Together with our previous results (Fig. 1) showing that *C. burnetii* levels were higher in STING^gt/gt^ BMDMs compared to WT BMDMs, these results suggest an inverse correlation between ROS levels and *C. burnetii* replication. To test this hypothesis, we treated WT BMDMs with the antioxidant N-acetyl cysteine (NAC) during *C. burnetii* infection. Indeed, the results showed that NAC-treated *C. burnetii*-infected WT BMDMs exhibit increased bacterial load at 12 dpi (Fig. S6A, B).

Previous reports suggest that ROS and calcium signaling have a mutual role in modulating each other’s function [60]. Furthermore, the mitochondrial depolarization induced by BAX is known to play a crucial role in triggering cell death and increased intracellular calcium levels [61]. In the context of infection, *Chlamydia trachomatis* has been reported to cause STING-dependent calcium signaling, leading to host cell death [62]. We hypothesized that calcium levels may be altered due to ROS production during *C. burnetii* infection in a STING-dependent manner. To investigate this, we treated infected BMDMs with a fluorescent Ca^2+^ indicator at 12 dpi and observed the cells using fluorescence microscopy. The results indicate that there are increased Ca^2+^ levels in infected WT BMDMs compared to STING^gt/gt^ BMDMs (Fig. 6E, F), aligning with our observed increase in ROS production and dysfunctional mitochondrial potential in *C. burnetii*-infected WT BMDMs compared to STING^gt/gt^ BMDMs.

Membrane depolarization due to BAX translocation to the mitochondria may be responsible for the escape of cytochrome c into the cytosol [61]. The presence of cytochrome c in the cytosol can activate caspase-9, which in turn activates caspase-3 leading to apoptosis [63]. Therefore, we investigated caspase-3 and -9 activation as a response to cytochrome c release during *C. burnetii* infection. Our results suggest that caspase-9 is cleaved during *C. burnetii* infection in a STING dependent manner, whereas infected STING^gt/gt^ BMDMs do not exhibit caspase-9 cleavage. Similarly, STING-dependent caspase-3 cleavage was observed in infected WT BMDMs (Fig. 6G).

Previous work has demonstrated the antiapoptotic effects of NAC-mediated ROS reduction through the inhibition of caspase-9 and caspase-3 activation [64–68]. Building on this, we explored the impact of NAC on caspase activation in *C. burnetii*-infected WT BMDMs. Our results show reduced cleavage of caspase-9 and caspase-3 in NAC-treated infected WT BMDMs at 12 dpi (Fig. S6C). This suggests a potential link between NAC-mediated reduction of ROS, modulation of apoptotic pathways, and the observed impact on bacterial survival during *C. burnetii* infection. We also investigated the effect of inhibiting BAX translocation to the mitochondria using the BAX translocation inhibitor peptide, humanin. This inhibition of BAX translocation with humanin decreased caspase-9 and caspase-3 activation in *C. burnetii*-infected BMDMs (Fig. S6D). Collectively, our results indicate that, during *C. burnetii* infection, STING activates mitochondrial dysfunction through BAX translocation to the mitochondria, leading to ROS production and increased intracellular calcium levels, which together lead to increased caspase-3 and -9 cleavage.

### Cytosolic mtDNA and 2′, 3′-cGAMP levels are decreased in *C. burnetii*-infected mouse BMDMs lacking STING

**Fig. 7:**
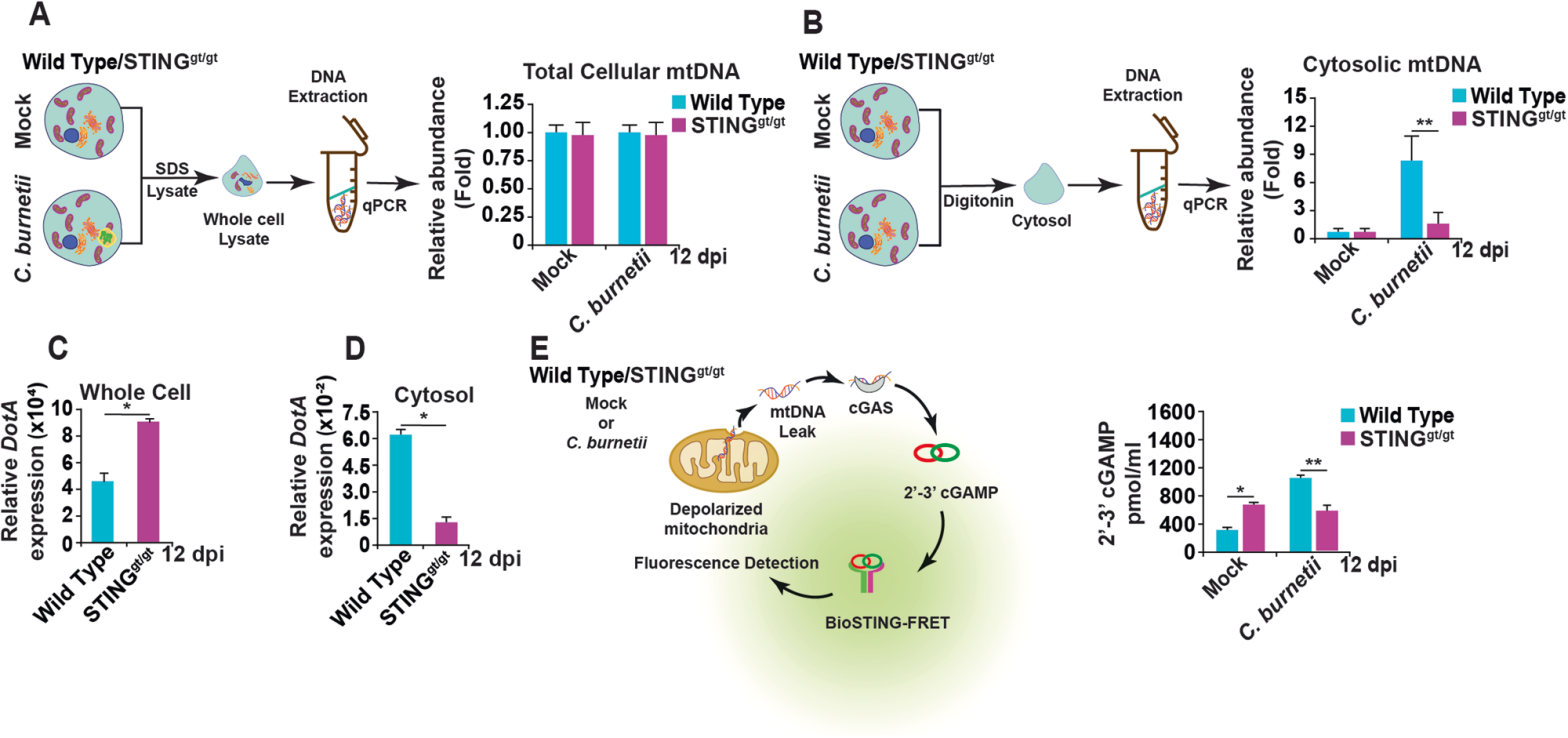
mtDNA detection in the cytosol of *C. burnetii-*infected BMDMs. **A** qPCR analysis of total cellular mtDNA (*D-loop*) normalized to total nuclear gene (*Tert*) in mock- and *C. burnetii*-infected WT and STING^gt/gt^ BMDMs at 12 dpi. **B** qPCR analysis of cytosolic mtDNA gene (*D-loop*) normalized to total cellular nuclear gene (*Tert*) in mock- and *C. burnetii*-infected WT and STING^gt/gt^ BMDMs at 12 dpi. **C** qPCR analysis of the *C. burnetii* gene (*DotA*) in whole cell lysate normalized to nuclear gene (*Tert*) from *C. burnetii*-infected WT and STING^gt/gt^ BMDMs at 12 dpi. **D** qPCR analysis of cytosolic *C. burnetii* gene (*DotA*) normalized to whole cell lysate nuclear gene (*Tert*) from infected *C. burnetii*-WT and STING^gt/gt^ BMDMs at 12 dpi. **E** 2′-3′ cGAMPs were detected in mock- and *C. burnetii*-infected WT and STING^gt/gt^ BMDMs at 12 dpi using the BioSTING-FRET assay. In (**A**–**E**) data are representative of four biological replicates from three independent experiments. Error bars, SEM. Unpaired *T*-test, *p < 0.05, **<0.01.

Translocation of BAX to the mitochondrial membrane causes pore formation that results in leakage of mtDNA into the cytosol [69, 70]. Our results show that STING activation leads to BAX translocation to the mitochondria causing depolarization during late-stages of *C. burnetii* infection. We next investigated the effects of mitochondrial membrane depolarization on mtDNA leakage during *C. burnetii* infection of WT and STING^gt/gt^ BMDMs at 12 dpi. Cells were lysed and separated into whole cell and cytosolic fractions, then total cellular mtDNA was analyzed using qPCR by probing for nuclear (*Tert*) and mitochondrial (*d-loop*) specific primers from whole cell fractions [71, 72]. There was no difference in the expression of *d-loop* relative to the nuclear gene *Tert* among mock- and *C. burnetii-*infected WT or STING^gt/gt^ BMDMs using whole cell lysates (Fig. 7A). However, the cytosolic fraction from *C. burnetii-*infected WT BMDMs exhibited increased expression of mitochondrial *d-loop* relative to nuclear *Tert* (Fig. 7B). These results suggest there is mtDNA in the cytosol of infected BMDMs in a STING-dependent manner.

We also investigated the presence of bacterial DNA in BMDMs during *C. burnetii* infection, both in whole cells and cytosolic fractions. While bacterial DNA was present at early stages of infection (Fig. S7A), by 6–12 dpi, the whole cell fraction, which contains CCVs, from WT BMDMs had a lower relative expression of the bacterial gene *DotA* compared to STING^gt/gt^ BMDMs (Fig. 7C). These results support those shown in Fig. 1, that in the absence of STING, *C. burnetii* is replicating to higher levels by later times post-infection. However, the cytosolic fraction of *C. burnetii*-infected STING^gt/gt^ BMDMs, which does not contain CCVs, exhibited lower bacterial DNA than WT BMDMs only at 12 dpi (Figs. 7D and S7B). Taken together, these results suggest that STING-dependent, BAX-mediated mitochondrial depolarization leads to mtDNA leakage into the cytosol of *C*. *burnetii-*infected WT BMDMs and that the cytosol of these cells contains increased bacterial DNA compared to cells lacking STING.

Cytosolic DNA during pathogenic infection or from leakage of host cell nuclear or mitochondrial DNA binds to cGAS, which then metabolizes 2′,3′-cGAMPs to activate STING and its downstream signaling [7]. The presence of increased mtDNA and bacterial DNA in the cytosol of *C*. *burnetii-*infected BMDMs in a STING-dependent manner prompted us to investigate the presence of 2′,3′-cGAMPs in these cells. *C. burnetii-*infected WT BMDMs had a higher 2′,3′-cGAMP concentration compared to infected STING^gt/gt^ BMDMs (Fig. 7E). These results suggest that a higher amount of cytosolic mtDNA and bacterial DNA in infected WT BMDMs leads to increased 2′,3′-cGAMPs concentration. Taken together, increased 2′,3′-cGAMPs at 12 dpi in a STING-dependent manner leads to feed-forward STING activation of its downstream signaling pathways, resulting in a cytochrome c mediated, caspase 9-activated form of apoptotic cell death.

### cGAS mediates STING activation during late-stage *C. burnetii* infection

**Fig. 8:**
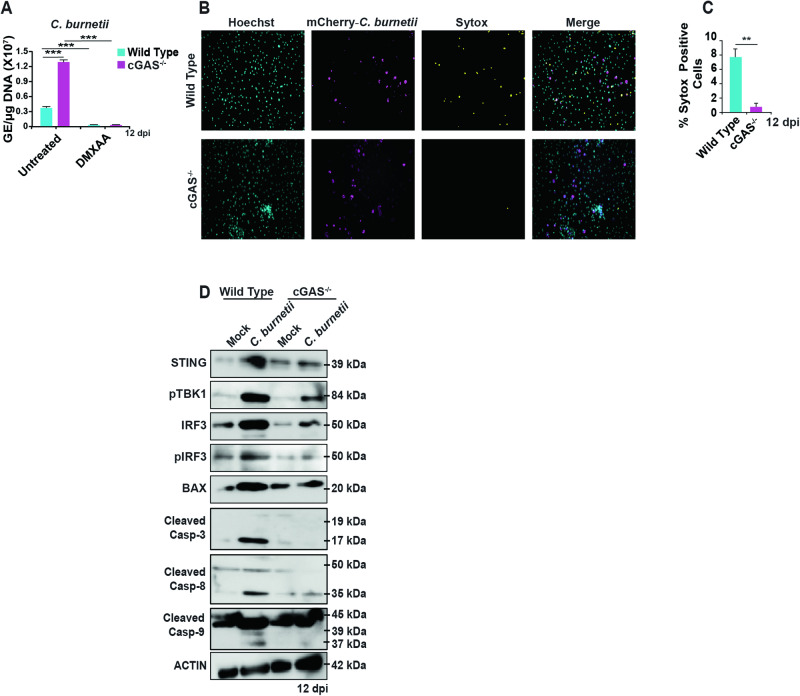
STING activation is mediated by cGAS during *C. burnetii* infection. **A** Bacterial load in WT and cGAS^−/−^ BMDMs at 12 dpi measured as genomic equivalents (GE) in the presence and absence of the pharmacological STING activator DMXAA (25 μg/ml). Data are representative of three biological replicates of 2 × 10^5^ cells per well of each genotype from at least three independent experiments. Error bars, SEM. Unpaired *T*-test, ***p < 0.001. **B** Fluorescent micrographs of mCherry-*C. burnetii*-infected (magenta) WT and cGAS^−/−^ BMDMs at 12 dpi, Hoechst 33342 (cyan) nuclear staining, and SYTOX (yellow) staining to detect dead cells. **C** Percentage of SYTOX-positive WT and cGAS^−/−^ BMDMs during *C. burnetii* infection at 12 dpi. Data are representative of three different fields of view from three independent experiments. Error bars, SEM. Unpaired *T*-test, **p < 0.01. **D** Western blot analysis of STING signaling components and caspases in mock- or *C. burnetii*-infected WT and cGAS^−/−^ BMDMs using the indicated antibodies at 12 dpi. Blots are representative of three independent experiments.

Since our model so far indicates the contribution of cGAS-generated 2′-3′ cGAMPs in the activation of STING during infection, we next determined the effect of cGAS on *C. burnetii* infection using WT and cGAS-deficient (cGAS^−/−^) BMDMs. Our results indicate that the absence of cGAS facilitates increased bacterial replication at 12 dpi, underscoring the critical role of cGAS in activating the STING signaling pathway (Fig. 8A). Nevertheless, in the absence of cGAS, treating cells with DMXAA resulted in inhibited bacterial replication, indicating that even in the absence of cGAS, STING activation can still effectively engage components of the STING signaling pathway (Fig. 8A). Next, we analyzed the role of cGAS on cell death during late-stage *C. burnetii* infection at 12 dpi. We observed that cell death was reduced at 12 dpi, in infected cGAS^-/-^ BMDMs compared to WT BMDMs (Fig. 8B, C).

Next, we examined the activation of STING signaling components in the presence and absence of cGAS during *C. burnetii* infection. At 12 dpi, the absence of cGAS led to a reduction in TBK1 and IRF3 phosphorylation, contrasting the heightened phosphorylation observed in infected WT BMDMs (Fig. 8D). Additionally, there was decreased caspase-3, -8, and -9 cleavage in cGAS^−/−^ BMDMs during *C. burnetii* infection (Fig. 8D). Collectively, our data strongly suggests that cGAS mediates STING activation during late-stage *C. burnetii* infection.

## Discussion

**Fig. 9:**
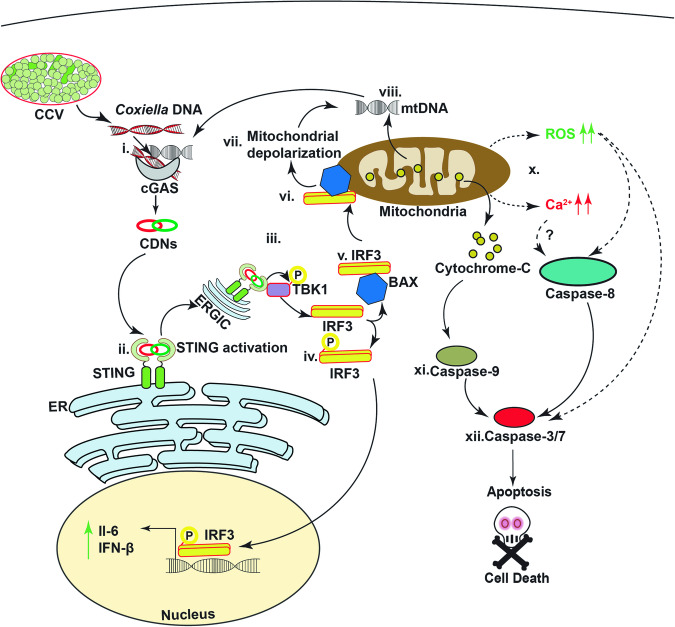
Model for STING mediated IRF3/BAX induced apoptosis during *C. burnetii* infection. Initial priming from *C. burnetii* DNA (i) activates STING signaling (ii) leading to its localization to ERGIC where phosphorylation of TBK1 and IRF3 occurs (iii). Activated IRF3 translocate to the nucleus to active type I IFNs and cytokines (iv). Cytosolic IRF3 complexes with BAX (v) stimulating its translocation to the mitochondrial membrane (vi) causing its depolarization (vii). mtDNA (viii) and cytochrome c (ix) leak out due to the mitochondrial damage. Cytosolic mtDNA then gets converted into 2′-3′ cGAMP by cGAS (i) making a positive feedback loop to intensify STING signaling causing more mitochondrial damage elevating intracellular reactive oxygen species and calcium levels (x). The cytochrome c in the cytosol activates caspase-9 (xi) which in turn cleaves procaspase 3 to activate caspase-3 (xii) leading to apoptosis.

In this study, we investigated how *C. burnetii* infection causes STING activation and leads to mitochondrial membrane depolarization followed by cell death. The recognition of cytosolic bacterial DNA by cGAS triggers STING activation, initiating IFNβ induction and setting up an amplification loop that increases STING levels. The elevated STING levels induce increased expression of BAX and IRF3, prompting their interaction. This STING mediated interaction leads to BAX translocation to the mitochondria, facilitating mtDNA leakage and initiating a cycle that reinforces STING activation via another positive feedback loop relayed through cGAS-generated cGAMPs. Furthermore, the absence of STING during *C*. *burnetii* infection allows for higher bacterial replication and reduced cell death due to decreased caspase activation. These experiments explain the mechanism of STING activation and its role in BAX-IRF3 induced mitochondrial mediated cell death during late-stage *C. burnetii* infection (Fig. 9).

Intracellular pathogens deploy various strategies to enhance their survival and avoid clearance from the host [73, 74]. Several studies show that *C. burnetii* infection arrests the apoptotic pathway in HeLa, CHO, and THP-1 cells during early-stage infection (1–2 dpi) [32, 37, 38]. While previous studies have focused on early times post-infection, in this study, we investigated host responses to *C. burnetii* during late-stage infection. Acute *C. burnetii* infection is often asymptomatic and self-limiting, but may progress into chronic infection leading to serious disease including endocarditis [29]. Polymorphisms within human populations can affect the immune responses, making some individuals more susceptible or tolerant to diseases [75]. Host factors are responsible for disease outcome, and immunocompromised individuals typically progress to chronic infection [76]. We have identified STING as a crucial host factor for *C. burnetii* infection, and its absence results in higher bacterial replication during late-stage infection of BMDMs. The effect of STING on bacterial replication is not cell type specific, however, as we show that pharmacological inhibition of STING with H-151 reduces bacterial load in *C. burnetii-*infected L929 mouse fibroblasts cells.

STING plays an essential role in nucleic acid recognition and induction of type 1 responses during viral infection [43, 77, 78]. Not only so, but intracellular bacteria such as *Listeria monocytogenes, Brucella abortus, Chlamydia muridarum*, and *Mycobacterium tuberculosis* induce a STING-dependent type I IFN response by early times post-infection [79–81]. While *C. burnetii* infection of mouse macrophages is immunologically silent at early times post-infection and does not induce type I IFNs [41], by late times post-infection, we observed IFNβ and IL-6 responses in a STING-dependent manner. However, the type of media used to differentiate BMDMs has an effect on the permissiveness of the avirulent NMII strain of *C. burnetii* [82]. Conditioned media, as used in this study, restricts NMII growth at early times post-infection, but the use of macrophage-colony stimulating factor (M-CSF) to differentiate BMDMs results in robust NMII growth. Future studies on the role of STING during *C. burnetii* infection should take this into account. Physiologically, acute Q fever patients show increased serological levels of TNFα and IL-6, whereas patients with valvopathy progressed to chronic infection have even higher amounts circulating TNFα and type I IFN, as well as reduced IL-1 receptor antagonist (IL-1Ra) [83, 84]. Similarly, we show that in the absence of STING at late times post-infection, there are higher TNFα levels and reduced *Il-1ra* induction as compared to control cells, suggesting that STING might have a role in the progression to chronic infection.

Previous studies suggest a role for STING in apoptosis independent of its role in inducing type I IFNs. For instance, in T cells and malignant B cells, the pharmacological activation of STING induces apoptosis [15]. It has also been shown that induction of type I IFN, specifically IFNβ, creates a positive feedback loop that enhances STING induction [46]. We found that the presence of leaked *C. burnetii* DNA in the cytosol serves as a trigger for cGAS, activating the STING pathway and culminating in the production of IFNβ. Consequently, this process amplifies STING expression levels during the late stages of infection, establishing a regulatory loop wherein STING activation triggers IFNβ production, further enhancing STING expression. These intensified levels of STING activation during late-stage *C. burnetii* infection leads to apoptosis induction. We show that the absence of STING reduces apoptosis due to caspase activation, resulting in increased bacterial load. However, in cells with reduced STING activation and apoptosis, increased overall bacterial load may also be due to non-apoptotic cell death that leads to released *C. burnetii* infecting nearby cells.

During viral infection, cytosolic IRF3 is induced to form a complex with BAX, which then translocates to the mitochondria leading to apoptosis [50–52]. Similarly, Liu et al. highlights a significant role of IRF3 in BAX recruitment to the mitochondria during RNA viral infections. Their findings suggest that IRF3 interacts with the mitochondrial proteins TOM70 and HSP90 to facilitate IRF3 translocation to mitochondria. [85]. Research examining the role of STING in ER stress during the initial stages of alcoholic liver disease has also provided evidence showcasing the presence of IRF3 within mitochondrial fractions at basal levels [55]. Similarly, STING-dependent elevated BAX and IRF3 levels during *C. burnetii* infection favor formation of the BAX/IRF3 complex, leading to BAX translocation to the mitochondria. BAX induces mitochondrial membrane permeabilization and the release of cytochrome c, which in turn can induce apoptosis [86–88]. We observed STING-dependent, BAX-mediated mitochondrial depolarization with the release of cytochrome c followed by the induction of caspase-3-mediated cell death during *C. burnetii* infection.

Mitochondrial stress and membrane depolarization leads to increased ROS and intracellular Ca^2+^ levels [89, 90]. Indeed, there is crosstalk between mitochondrial depolarization, ROS, and calcium homeostasis [91]. STING regulates ROS homeostasis, and the absence of STING reduces ROS, affecting susceptibility to radiation-induced cell death in tumor cells [92]. We observed increased ROS levels during *C. burnetii* infection due to mitochondrial depolarization, which interferes with calcium regulation in a STING-dependent manner. *Chlamydia trachomatis* or viral infection has been shown to induce cell death in a STING-dependent manner which relies on increased intracellular calcium levels [62, 93]. Similarly, we show that the absence of STING during *C. burnetii* infection reduces intracellular calcium levels and cell death. Together, mitochondrial depolarization causes cytochrome c release, which then drives cells toward apoptotic cell death, relayed by elevated ROS and intracellular Ca^2+^ induction during *C. burnetii* infection in a STING-dependent manner.

Mitochondrial membrane depolarization, ROS induction, and increased intracellular ROS, exerts stress on mitochondria that leads to mtDNA leakage into the cytosol [69, 94–96]. During pathogenic infection, cytosolic mtDNA presence due to mitochondrial stress induces expression of STING and induction of type I IFNs [97, 98]. Mitochondrial stress and mtDNA leakage is a major contributor of IFNβ production [99]. Indeed, we detected mtDNA leakage into the cytosol in a STING-dependent manner during *C. burnetii* infection.

We hypothesize that initial priming of STING activation during *C. burnetii* infection is caused by the presence of low amounts of bacterial DNA in the cytosol, which is converted into cGAMP early during infection, leading to a positive feed-forward loop involving IFNβ production causing increased STING expression. In turn, this STING activation leads to BAX/IRF3 mediated mtDNA leak from depolarized mitochondria that maintains sustained STING activation, causing intensified STING signaling that stimulates mitochondria-mediated cell death. The heightened activation of STING, whether triggered by sterile activation or pathogenic stimuli, can lead to lysosomal damage by compromising membrane integrity, potentially resulting in cell death [19, 100]. Elevated levels of intracellular ROS and calcium, which are known consequences of STING activation, have been identified as factors detrimental to lysosomal stability [101–103]. The STING-dependent increase in intracellular ROS and calcium levels we observed might contribute to the permeabilization of the CCV, causing its leakage and exposing *C. burnetii* to the cytosol. Our findings support this notion, as infected WT BMDMs exhibited higher levels of bacterial DNA in the cytosol than STING-deficient BMDMs during late-stages of infection.

In conclusion, we have demonstrated that STING signaling is critical for BAX-IRF3-mediated, mitochondrially induced apoptosis during late-stages of *C. burnetii* infection. Additionally, induction of higher TNFα and reduced IL-1Ra in the absence of STING may be involved in the progression to chronic disease. Agonist-mediated activation of STING has been documented to contribute to the efficacy of various cancer and immunotherapies [104, 105]. Therefore, we propose that activation of STING with agonists to stimulate programmed cell death pathways may work in combination with existing antibiotic treatments to effectively control progression of *C. burnetii* infection to chronic disease.

## Experimental procedures

### Cell culture

BMDMs were isolated from 6- to 8-week-old female C57BL/6 (Jackson Labs 000664), STING^gt/gt^ mouse (Jackson Labs 017537) and cGAS KO mouse (Jackson Labs 026554) femurs received from the Jackson laboratory as previously described [81, 106]. Briefly, BMDMs were differentiated from bone marrow cells using 30% L929 conditioned culture media (30% LCCM) supplemented with 1x DMEM and 10% fetal bovine serum (FBS, Hyclone), 1x antibacterial and antimycotic at 37 °C in 5% CO_2_. The bone marrow cells were incubated at 37 °C in 5% CO_2_ for 5 days and media was replaced with fresh bone marrow differentiation media (30% LCCM, 10% FBS and 1x antibacterial and antimycotic). L929 cells were grown in 1x DMEM supplemented with 10% FBS (Atlas Biologicals), 1x antibacterial and antimycotic at 37 °C in 5% CO_2_.

### Bacterial infections

*C. burnetii* NMII (clone 4 RSA439) was propagated in Acidified Citrate Cysteine Medium 2 (ACCM 2) as previously described [107]. *C. burnetii* NMII stocks were quantified using quantitative PCR (qPCR) to measure bacterial genome equivalent (GE), as previously described [27]. Differentiated WT and STING^gt/gt^ BMDMs were incubated with *C. burnetii* NMII or mCherry expressing *C. burnetii* NMII (mCherry-*C. burnetii* NMII) at MOI of 100 GE/cell for 1 h in infection media (1x DMEM, 2% FBS, 10% LCCM) at 37 °C in 5% CO_2_. The cells were washed three times with incomplete DMEM to remove extracellular bacteria (day 0) and media was replaced with 1x DMEM, 10% FBS, 10% LCCM. The L929 cells treated or untreated with H-151 (2 μM) STING inhibitor and DMXAA (25 μg/ml) STING activator was incubated with *C. burnetii* NMII or mCherry-*C. burnetii* at MOI of 300 GE/cell for 16 h (1x DMEM and 10% FBS) at 37 °C in 5% CO_2_. Twenty-four hours later, the cells were washed three times with incomplete DMEM to remove extracellular bacteria (day 0) and media was replaced with 1x DMEM and 10% FBS.

### Measurement of bacterial replication by genome equivalents

Quantification of *C. burnetii* from lysed BMDMs was performed as described in [108]. Briefly, bacterial DNA was released from cells by harvesting 5 × 10^5^ cells per condition to a single tube containing 0.3 ml of molecular grade water. The supernatant was transferred to a fresh tube containing 0.1 mm zirconia beads, homogenized three times at 5.0 m s^−1^ for 30 s, and centrifuged for 1 min at 12,000 × *g*. The homogenized lysate was quantitated for DNA using spectrophotometer (Biotek Cytation 3) 100 ng of DNA in molecular grade water was used for qPCR quantification.

### Cell viability assay

In total, 1 × 10^4^ cells per well were plated in 96-well plate and mock-infected or infected with *C. burnetii* at MOI of 100 GE/cell. At 12 dpi the cells were treated with a SYTOX Green Nucleic Acid Stain (Invitrogen S7020) according to the manufacturer’s instructions and live fluorescence microscopy was performed at ×10 magnification. Images were quantified using ImageJ software [109, 110].

### ELISA

WT and STING^gt/gt^ BMDMs were seeded at a density of 2 × 10^5^ cells/well in 12-well plates and infected with *C. burnetii* at 100 GE/cell. The cell-free supernatants (500 µl) were collected at 12 dpi from mock- and *C. burnetii*-infected WT and STING^gt/gt^ BMDMs. The supernatants were diluted with assay buffer as per manufacturers protocol and analyzed for the presence of IFNβ (Invitrogen 424001), IL-6 (Invitrogen KMC0061), and TNF-α (Invitrogen BMS607-2HS) using an enzyme-linked immunosorbent assay (ELISA) utilizing paired antibodies according to manufactures protocol.

### Flow cytometry

Annexin V binding buffer, fluorochrome-conjugated Annexin V, and propidium iodide (Invitrogen A35110) were used according to manufacturer’s instructions. The cells were detached using StemPro Accutase (Invitrogen A1110501) cell dissociation reagent to ensure cell maximum cell viability and cells were washed once with PBS and once with Annexin V binding buffer. Cells were stained with Annexin V (1:200 dilution) for 15 min at room temperature in Annexin V binding buffer. Caspase activation was evaluated as the percentage of cells with active caspase 3 and 8, using FAM-FLICA in vitro caspase detection kit (BioRad ICT093 and ICT910), used according to the manufacturer’s instructions. Data were acquired using Easycyte Guava flow cytometer and analyzed using Guava soft software.

### Mitochondria isolation

To isolate pure organelle fraction from *C. burnetii* infected L929 mouse fibroblasts, the cells were treated with or without H-151 (2 μM) STING inhibitor and infected with *C. burnetii* at MOI of 300 GE/cell. After 12 days-post infection cells were harvested using StemPro Accutase (Invitrogen A1110501) cell dissociation reagent. The cells were washed twice with 1X PBS at 300 × *g* for 5 min at 4 °C. After washing once with PBS, cells were washed with 1 ml 0.9% sodium chloride solution at 300 × *g* for 5 min at 4 °C. The pellet was resuspended in lysis buffer provided in the kit and cells were fractionated using Qproteome-mitochondria isolation kit according to manufacturer’s instructions (Qiagen 37612).

### Immunoblotting

Protein extracts were prepared by lysing cells with RIPA buffer (25 mM Tris-HCl pH 7.6, 150 mM NaCl, 1 mM EDTA, 1% NP-40, 1% sodium deoxycholate, 0.1% SDS, 1 mM Na3VO4, 1 mM NaF, 0.1 mM PMSF, 10 μM aprotinin, 5 μg/ml leupeptin, 1 μg/ml pepstatin A). Protein samples were diluted using 10x Laemmli loading buffer, mixed, and boiled for 5 min at 95 °C. Samples were analyzed by SDS/PAGE using a 12% and 15% acrylamide gel, followed by transfer onto PVDF membranes (Millipore IPVH00010). Membranes were blocked with 5% nonfat dry milk (Lab Scientific M0841) or 5% BSA (Thermo Fisher BP9706) in Tris-buffered saline (50 mM Tris-HCl pH 7.5, 150 mM NaCl) and 0.1% Tween-20 for 1 h at room temperature. Primary antibody labeling was completed with anti-caspase-3 (1:1000; Cell Signaling 9662), anti-cleaved caspase-3 (1:1000; Cell Signaling 9664), anti-Caspase-8 (1:1000; Proteintech 13423-1-AP), anti-cleaved caspase-9 (1:1000; Cell Signaling 20750), anti-STING (1:200; Sigma MABF213), anti-P-TBK1 (1:1000; Cell Signaling 5483), anti-IRF3 (1:1000; Cell Signaling 4302), anti-p-IRF3 (1:1000; Cell Signaling 4947), anti-BAX (1:500; Cell Signaling 2772), anti-cytochrome c (1:500; Cell Signaling 11940), anti-COXIV (1:1000; Cell Signaling 4850), anti-VDAC1 (1:1000; Cell Signaling 4661) anti-HistoneH3 (1:1000; Cell Signaling 60932), anti-GAPDH (1:1000; Cell Signaling 5174), or anti-actin (1:10,000; Sigma A5441) overnight at 4 °C. Secondary antibody labeling was completed using anti-rabbit IgG-HRP conjugate (1:10,000; Promega W401B) or anti-mouse IgG-HRP conjugate (1:10,000; Promega W402B) by incubating membranes for 1 h at room temperature. Blots were imaged onto film using SuperSignal West Pico Chemiluminescent Substrate (Thermo Fisher 34579).

### Immunoprecipitation

L929 cells treated with or without STING inhibitor H-151 (2 μM) (MedChemExpress HY-112693) and activator DMXAA (25 μg/ml) (MedChemExpress HY-10964) were infected with *C. burnetii* NMII. L929 cells were lysed after 12 days post infection using IP lysis buffer (Thermo Scientific 87787) with protease inhibitors cocktail (Thermo Scientific 78425). For IRF3 Pull-down: ~250 µg protein was incubated with 20 µl of appropriate suspended (25% v/v) agarose conjugated anti-IRF3 (Santa Cruz Biotechnology sc-33641) overnight at 4 °C on rotating nutator. Bead-protein complexes were washed 3 times in IP lysis buffer and then incubated 10 min at 97 °C with 2X loading dye. Lysates were centrifuged 5 min at 13,000 × *g* to remove the beads and subjected for western blotting analyses.

### Immunofluorescence microscopy

Mock- or *C. burnetii*-infected WT BMDM and STING^gt/gt^ were seeded onto coverslips in 8-well iBIDI chamber slides at a confluency of ~9 × 10^4^ cells/well. At 12 dpi cells were fixed in 4% paraformaldehyde for 10 min at room temperature, permeabilized in 0.1% Triton X-100 for 15 min at room temperature and blocked in 2% FBS in TBS for 45 min at 37 °C. Primary antibody labeling was completed with anti-STING (1:50), anti-IRF3 (1:100) and anti-BAX (1:100) overnight in a humified chamber at 4 °C. Secondary antibody labeling was completed using anti-rabbit (Life Technologies A11034 or A11035) or anti-mouse (Life Technologies A11029 or A11030) Alexa Fluor 488 or 546 (1:300) by incubating membranes for 1 h at room temperature in the dark. Samples were stained with DAPI (1:100; Cell Signaling 4083), mounted onto coverslips using ProLong Diamond Antifade Mount (Invitrogen P36961), and imaged using a Leica DMi8 fluorescence microscope. For mitochondrial staining 100 nM MitoTracker Red CMXRos (Invitrogen M7512) was treated to mock or infected BMDMs and incubated at 37 °C in 5% CO_2_ for 15 min followed by washing with incomplete DMEM. After staining with Mitotracker dye cells were fixed with protocol used above. Leica Application Suite X Imaging and Analysis Software was used to process the final images.

### Mitochondrial membrane potential assay

*C. burnetii* infection induced changes in infected BMDMs mitochondrial membrane potential (ΔΨm) were assessed using the fluorescent reagent tetraethyl benzimidazolyl carbocyanine iodide (JC-1) (Invitrogen T3168) following the manufacturer’s protocol. The cells were seeded at a density of 5 × 10^4^ cells/well and allowed to adhere overnight in a black, clear-bottom 96-well plate. The BMDMs WT and STING^gt/gt^ were infected with *C. burnetii* at 100 GE/cell. After 12 dpi BMDMs were treated 2 µM JC-1 dye for 10 min at 37 °C in 5% CO_2_, protected from light. JC-1 dye was then removed, cells were washed once with 1× incomplete DMEM (Gibco 11965092), 100 µl of fresh 1× FluoroBrite DMEM buffer (Gibco A1896701) was added to each well. JC-1 is cationic fluorescent dye which forms aggregates in the functional mitochondria (red fluorescence; shown as pseudo color magenta) with normal membrane potential whereas it is retained as monomer in dysfunctional mitochondria with depolarized membrane (green fluorescence; shown as pseudo color yellow). JC-1 dye aggregates show excitation and emission at 535 nm and 595 nm whereas monomer show excitation and emission at 485 nm and 535 nm, respectively.

The JC-1 treated BMDMs were then visualized with Biotek Cytation 3 imaging plate reader. The images were quantified using ImageJ and the ratio of magenta (normal potential) fluorescence divided by that of yellow (depolarized) fluorescence demonstrates the state of mitochondrial function of cell population.

### DCFDA (2′,7′-dichlorofluorescin diacetate) assay

Mock or Infected BMDMs were seeded in a black, clear bottom 96-well microplate at a cell density of 5 × 10^4^ cells/well. The cells were assayed using the DCFDA Cellular ROS Detection Assay reagent (Sigma 4091-99-0). Cells were analyzed using fluorescence microscope and images were captured at excitation and emission wavelengths of 485 and 535 nm, respectively. The images were quantified with ImageJ using Hoechst 33342 (Thermo Scientific 62249) nuclear stain to count the total number of cells.

### N-Acetyl-L-cysteine treatment assay

WT and STING^gt/gt^ BMDMs were pre-incubated with 2 mM N-acetyl-l-cysteine (NAC) (Sigma-Aldrich, A7250) for 16 h and were then infected with mCherry-*C. burnetii* at 100 GE/cell. During media replacement everyday 2 mM N-acetyl-l-cysteine (NAC) was added to mock and mCherry-*C*. *burnetii* infected WT and STING^gt/gt^ BMDMs. At 12 dpi, mock and mCherry-*C*. *burnetii* infected cells were analyzed using fluorescence microscope and images were captured at excitation and emission wavelengths of 587 and 610 nm, respectively. The images were quantified for mCherry-*C*. *burnetii* positive cell population with ImageJ using Hoechst 33342 (Thermo Scientific 62249) nuclear stain to count the total number of cells. Lysate collected from NAC treated WT and STING^gt/gt^ BMDMs infected with mCherry-*C*. *burnetii* at 100 GE/cell at 12 dpi for western blot analysis.

### Intracellular calcium detection

To detect the intracellular calcium levels during infection of BMDMs with *C. burnetii* NMII, the WT and STING^gt/gt^ BMDMs were seeded in a black, clear bottom 96-well microplate at a cell density of 4 × 10^4^ cells/well infected at MOI of 100. After 12 days post infection the intracellular calcium levels were detected in mock and infected BMDMs using Fluo-4 NW Calcium Assay Kit according to manufactures instructions (Invitrogen F36206). Cells were analyzed using fluorescence microscopy and images were captured with excitation at 494 nm and emission at 516 nm, respectively. The images were quantified with ImageJ using Hoechst 33342 (Thermo Scientific 62249) nuclear stain to count the total number of cells.

### Cytosolic mtDNA detection

To detect the mitochondrial DNA in the cytosol of *C. burnetii* infected BMDMs, the cells were seeded at 1 × 10^6^ cells/well for at least 24 h. The WT and STING^gt/gt^ BMDMs were infected with mock and *C. burnetii* at 100 GE/cell. After 12 dpi the mock and infected BMDMs were harvested and lysed for the fractionation into the cytosolic extract and whole cell extracts as previously described [71, 72]. DNA isolated from cytosolic and whole cell extract was used for detecting mtDNA.

### Quantitative reverse transcriptase PCR

Total RNA was isolated using the Direct-Zol RNA miniprep kit (Zymo research R2052) and cDNA was synthesized using iScript Reverse Transcription Supermix (BioRad 1708840). Quantitative RT-qPCR was performed in duplicate using single tube TaqMan assay (*Gapdh, Ifnb, Il6, Il1b, Tnfa* and *Tmem173)* and Maxima SYBR Green Master Mix (*Tert, dloop* and *DotA*) (Thermo Scientific K0221). For all gene expression data *Gapdh* was used as an endogenous normalization control. Taqman assays: *Gapdh* assay id: Mm00434228_m1*, Ifnb1* assay id: Mm00439552_S1, *Il6* assay id: Mm00446190_m1, *Tnfa* assay id: Mm00443258_m1, and *Il1b* assay id: Mm00434228_m1. Primer sequences are as follows: Mm*Tert* forward, 5′- CTAGCTCATGTGTCAAGACCCTCTT-3′; Mm*Tert* reverse, 5′- GCCAGCACGTTTCTCTCGTT-3′; mt*Dloop* forward, 5′- AATCTACCATCCTCCGTGAAACC -3′; mt*Dloop* reverse, 5′- TCAGTTTAGCTACCCCCAAGTTTAA -3′. *C. burnetii DotA* forward, *5*′*- CTAGCTCATGTGTCAAGACCCTCTT-3*′; *C. burnetii DotA* reverse, 5′- GCCAGCACGTTTCTCTCGTT-3′.

### 2′,3′-cGAMP measurements

To measure intracellular 2′,3′-cGAMPs 1 × 10^6^ BMDMs were infected with *C. burnetii* at 100 GE/cell. At 12 dpi mock and infected WT and STING^gt/gt^ BMDMs were harvested, and pellets were incubated on ice with pre-chilled 200 μl 80% methanol for 20 min (Pollock et al. [111]). After incubation in methanol cells were lysed using sonication and the lysed extracts were concentrated using speed vac. The dried cell extracts were reconstituted with the 1x assay buffer. The Relative concentration of 2′,3′-cGAMPs measurements were quantified using 2′,3′-Cyclic GAMP STING-Based FRET Detection Kits (Arbor Assays K081).

### Quantification and statistical analyses

Statistical analyses were completed using GraphPad Prism. Two-tailed unpaired *T-*tests assuming unequal variance, with multiple comparisons were utilized to compare normally distributed pairwise quantitative data of at least three independent experiments, as specified in the figure captions. Images and Pearson’s correlation coefficient (PCC) were quantified with ImageJ software using four different micrographs. All error bars represent standard error of the mean.

### Supplementary information

Combined supplementary file

Uncropped Western blots

Reproducibility checklist

## Data Availability

The datasets generated during and/or analyzed during the current study are available from the corresponding author on reasonable request. Western blot data generated or analyzed during this study are included in this published article and its Supplementary Information files.
