# Supplementary material for: STING dependent BAX-IRF3 signaling results in apoptosis during late-stage Coxiella burnetii infection
Source: Cell Death Dis. 2024 Mar 8;15(3):195. doi: 10.1038/s41419-024-06573-1 (PMC10924102; doi:10.1038/s41419-024-06573-1)
Supplement: Supplementary file 1 — Combined supplementary file [file 41419_2024_6573_MOESM1_ESM.pdf]

STING dependent BAX-IRF3 signaling results in apoptosis during late-stage *Coxiella burnetii* infection.

Manish Chauhan[1], Chelsea A. Osbron[1], Heather S. Koehler[1], and Alan G. Goodman[1,2]*

[1]School of Molecular Biosciences, College of Veterinary Medicine, Washington State University, Pullman, WA 99164, USA

[2]Paul G. Allen School for Global Health, College of Veterinary Medicine, Washington State University, Pullman, WA 99164, USA

* Correspondence: alan.goodman@wsu.edu

Supplementary Figures 1-7 and legends.

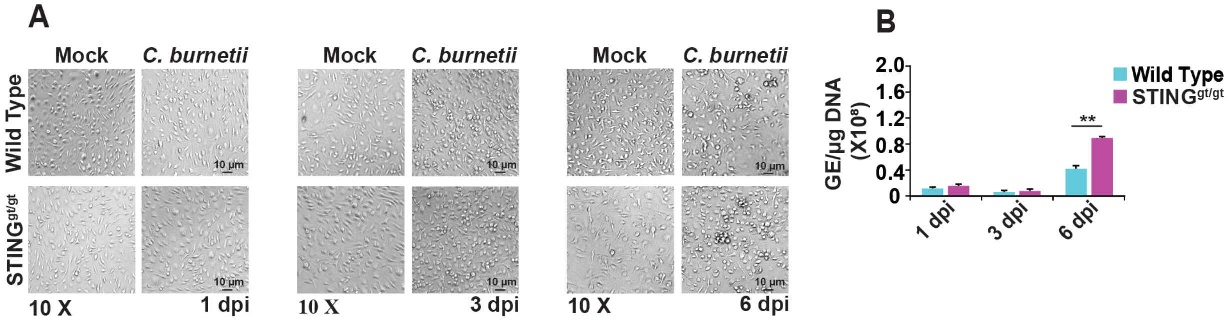

Supplementary Figure 1 (related to Figure 1). STING deficiency leads to increased bacterial load.
(A) Differential Interference Contrast (DIC) micrograph of mock- and *C. burnetii*-infected WT and STING$^{gt/gt}$ BMDMs at the indicated times. Mircographs are representative of three independent experiments.
(B) Bacterial load in WT and STING$^{gt/gt}$ BMDMs at indicated dpi measured as genomic equivalents (GE). Data are representatve of three biological replicates each from three independent experiments. Error bars, SEM. Unpaired T test, **p<0.01

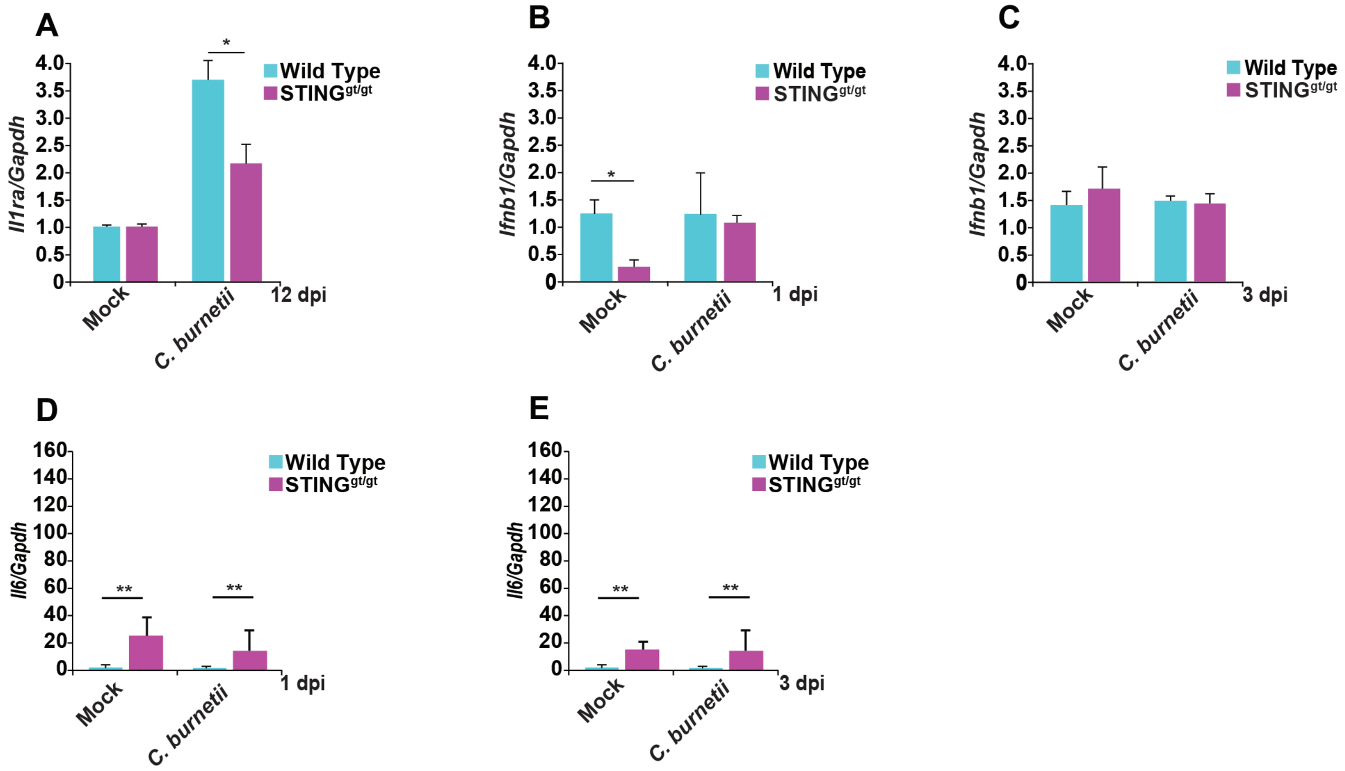

Supplementary Figure 2 (related to Figure 2). Host response to *C. burnetii* infection in the presence and absence of STING

(A) qPCR for detection of *Il1ra* in mock- and *C. burnetii*-infected WT and STING$^{gt/gt}$ BMDMs at 12 dpi. Data are representatve of four biological replicates each from three independent experiments. Error bars, SEM. Unpaired T test, *p<0.05.

(B-C) qPCR for detection of *ifnb1* in mock- and *C. burnetii*-infected WT and STING$^{gt/gt}$ BMDMs at 1 and 3 dpi. Data are representatve of three biological replicates each from three independent experiments. Error bars, SEM. Unpaired T test, *p<0.05.

(D-E) qPCR for detection of *Il6* in mock- and *C. burnetii*-infected WT and STING$^{gt/gt}$ BMDMs at 1 and 3 dpi. Data are representatve of three biological replicates each from three independent experiments. Error bars, SEM. Unpaired T test, **p<0.01

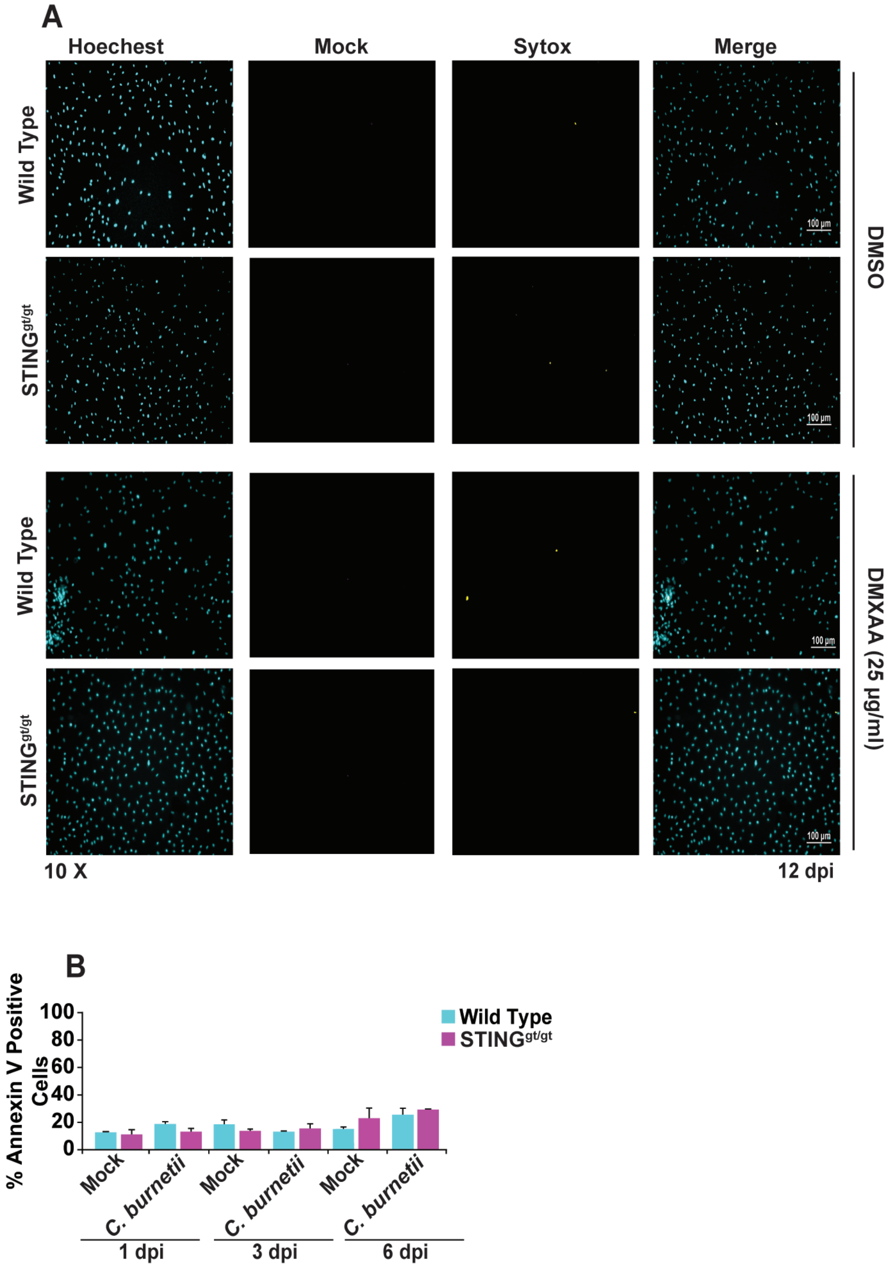

Supplementary Figure 3 (related to Figure 3). STING promotes programmed cell death during *C. burnetii* infection in BMDMs.

(A) Fluorescent micrographs of mock-infected (magenta) WT and $STING^{gt/gt}$ BMDMs in presence and absence of DMXAA at 12 dpi, nuclear staining dye Hoechst 33342 (cyan) and SYTOX (Yellow) dye to detect dead cells.The Micrographs are representative of at least three independent experiments.

(B) Percentage of Annexin V positive cells from mock- and *C. burnetii*-infected WT and $STING^{gt/gt}$ BMDMs at 1, 3, and 6 dpi. Data are representative of three biological replicates from three independent experiments. Error bars, SEM. Unpaired T test.

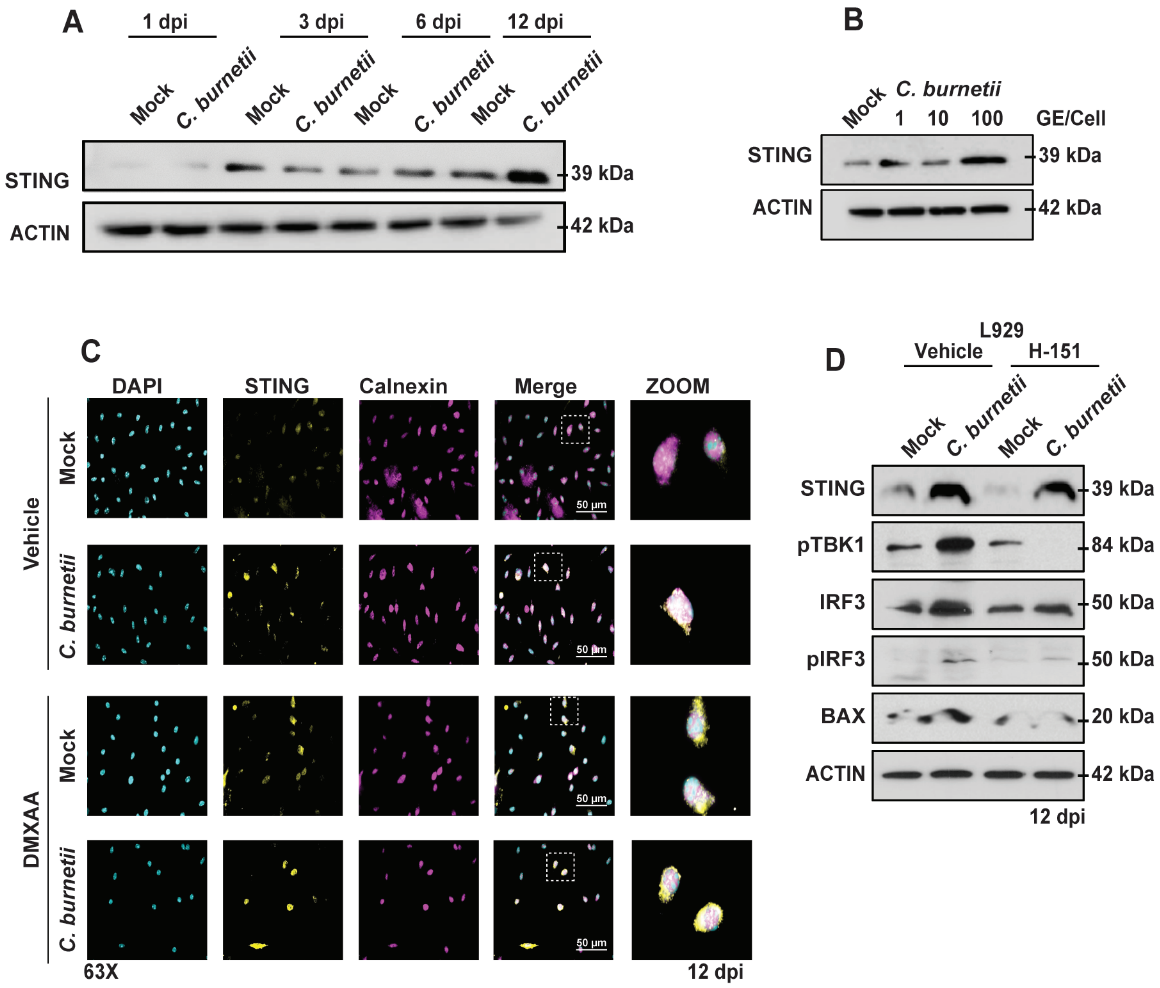

Supplementary Figure 4 (related to Figure 4). Elevated STING levels during *Cb* infection induce mitochondria-mediated apoptosis through the BAX-IRF3 pathway.

(A) Western blot analysis of STING levels during mock- or *C. burnetii*-infected Wild Type BMDMs at 1,-3,-6 and -12 dpi.

(B) Western blot analysis of STING levels during mock- or *C. burnetii*-infected Wild Type BMDMs at various MOIs (indicated on blot) at 12 dpi.

(C) Micrographs show colocalization of STING (yellow) and Calnexin (magenta) in mock- or *C. burnetii*-infected WT BMDMs in the presence and absence of DMXAA at 12 dpi. The micrographs are representative of three independent experiments.

(D) Western blot analysis of STING signaling components in mock- or *C. burnetii*-infected L929 cells treated with vehicle or H-151 (2 µM) using the indicated antibodies at 12 dpi.

All blots are representative of three independent experiments.

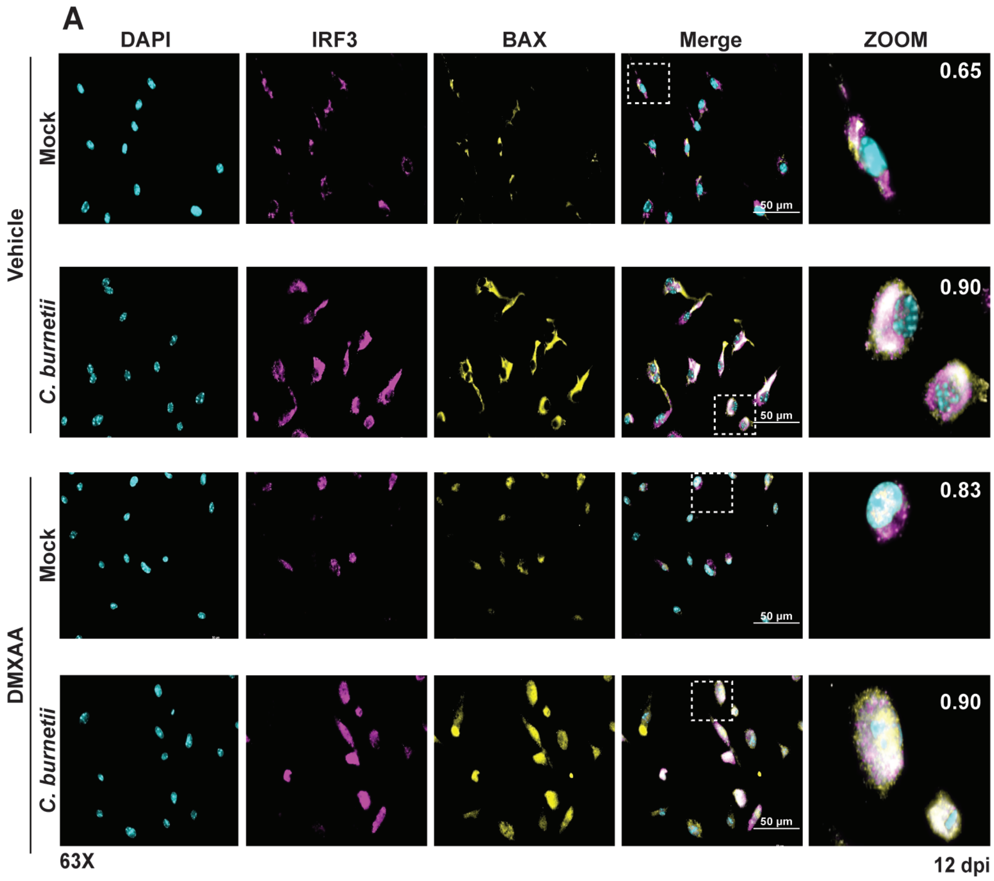

Supplementary Figure 5 (related to Figure 5). STING activation induces BAX/IRF3 colocalization
(A) Micrographs show colocalization of IRF3 (magenta) and BAX (yellow) in mock- or *C. burnetii*-infected WT BMDMs in presence and absence of DMXAA at 12 dpi. Mircographs are representative of four independent expereiments. Pearson's correlation coefficient for localization overlap is mentioned in white numeric font in zoomed out micrograph.For Pearson's correlation coefficient quantitation four different micrographs were analyzed (N=4).

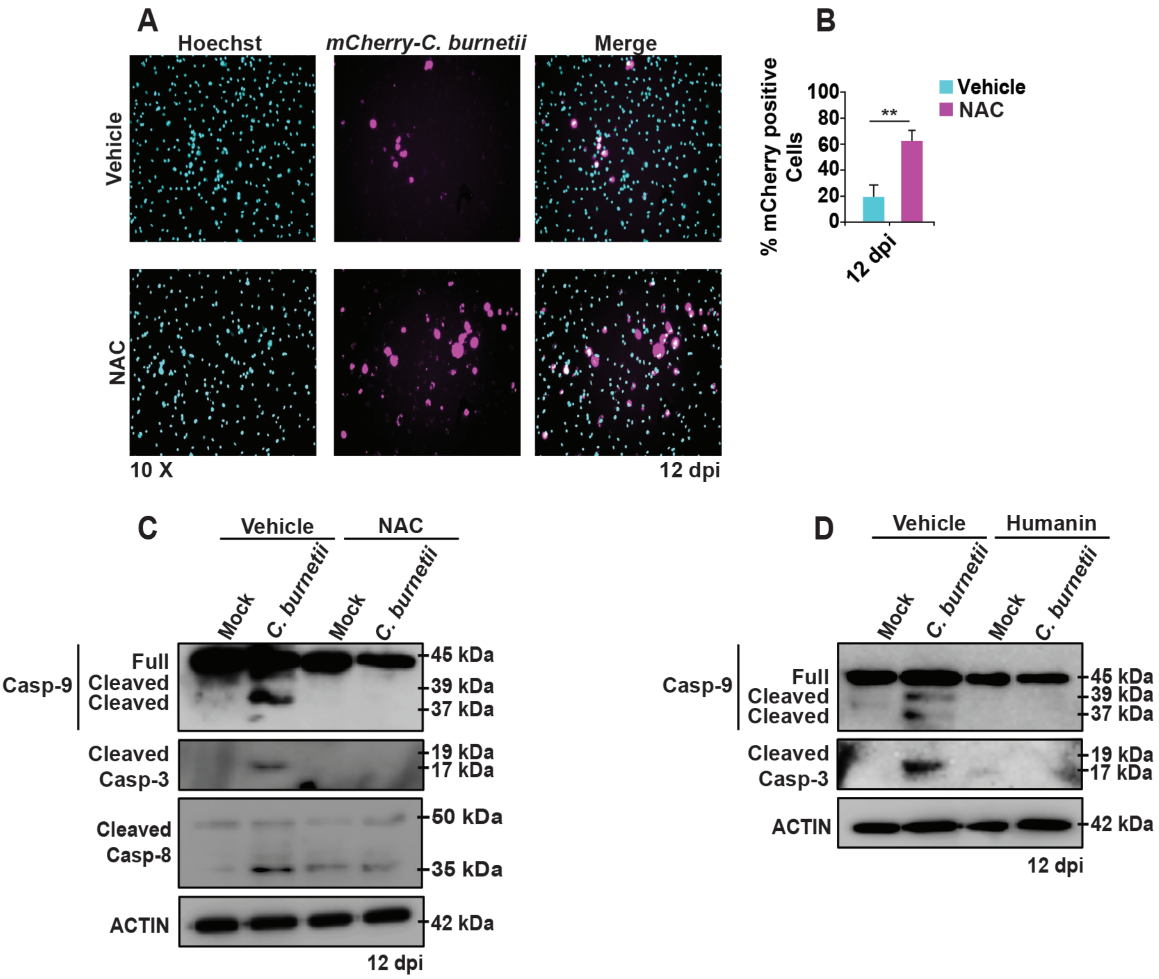

Supplementary Figure 6 (related to Figure 6). STING activation during *C. burnetii* infection induces mitochondrial depolarization leading to increased ROS and calcium levels in mouse BMDMs.

(A) Fluorescent micrographs show mCherry-*C. burnetii* infected (magenta) WT BMDMs in the presence and absence of NAC (N-Acetyl Cysteine) 2 mM and counterstained with nuclear staining dye Hoechst 33342 (cyan).

(B) Percentage of mCherry-positive WT BMDMs in the presence and absence of NAC (N-Acetyl Cysteine) 2mM and counterstained with nuclear staining dye Hoechst 33342 (cyan) at 12 dpi. Data are representative of five different fields of view from three biological replicates of each genotype from three independent experiments. Error bars, SEM. Unpaired T test, **p<0.01.

(C) Western blot analysis of mock- or *C. burnetii*-infected WT BMDMs in the presence and absence of NAC (N-Acetyl Cysteine) 2 mM using the indicated antibodies at 12 dpi. All blots are representative of three independent experiments.

(D) Western blot analysis of mock- or *C. burnetii*-infected WT BMDMs in the presence and absence of Humanin (BAX mitochondrial translocation inhibitor) 10 μM using the indicated antibodies at 12 dpi. All blots are representative of three independent experiments.

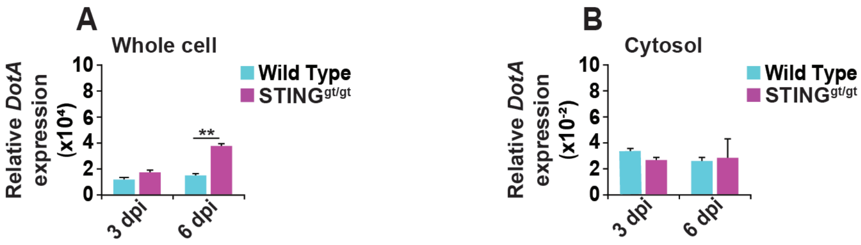

Supplementary Figure 7 (related to Figure 7) . mtDNA and bacterial DNA detection in the cytosol of *C. burnetii* infected BMDMs

(A) qPCR analysis of *C. burnetii* gene (*DotA*) in whole cell lysates normalized to the nuclear gene (*Tert*) in *C. burnetii*-infected WT and STING$^{gt/gt}$ BMDMs at 3 and 6 dpi.

(B) qPCR analysis of cytosolic *C. burnetii* gene (*DotA*) normalized to whole cell lysate nuclear gene (*Tert*) in *C. burnetii*-infected WT and STING$^{gt/gt}$ BMDMs at 3 and 6 dpi.

In A & B data is representative of three biological replicates from three independent experiments. Error bars, SEM. Unpaired T test, **<0.01.
