# Supplementary material for: STING dependent BAX-IRF3 signaling results in apoptosis during late-stage Coxiella burnetii infection
Source: Cell Death Dis. 2024 Mar 8;15(3):195. doi: 10.1038/s41419-024-06573-1 (PMC10924102; doi:10.1038/s41419-024-06573-1)
Supplement: Supplementary file 2 — Uncropped Western blots [file 41419_2024_6573_MOESM2_ESM.pdf]

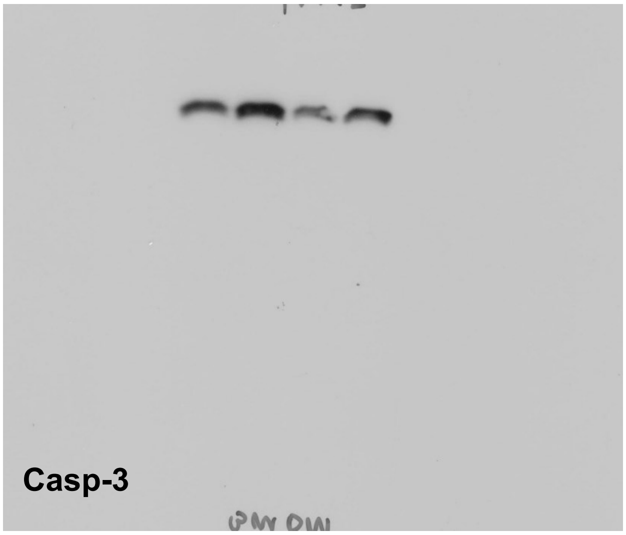

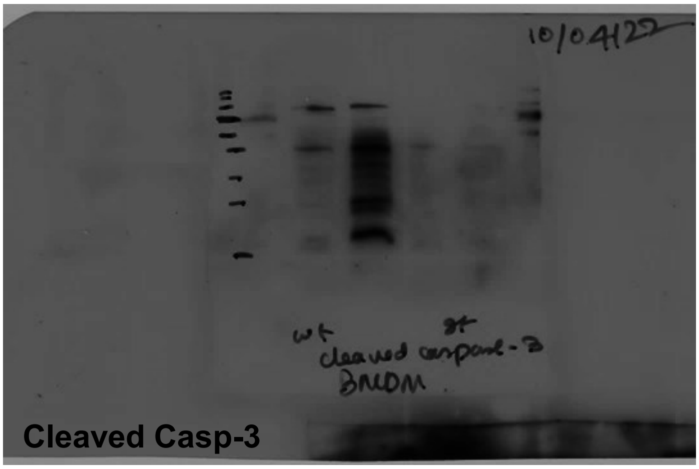

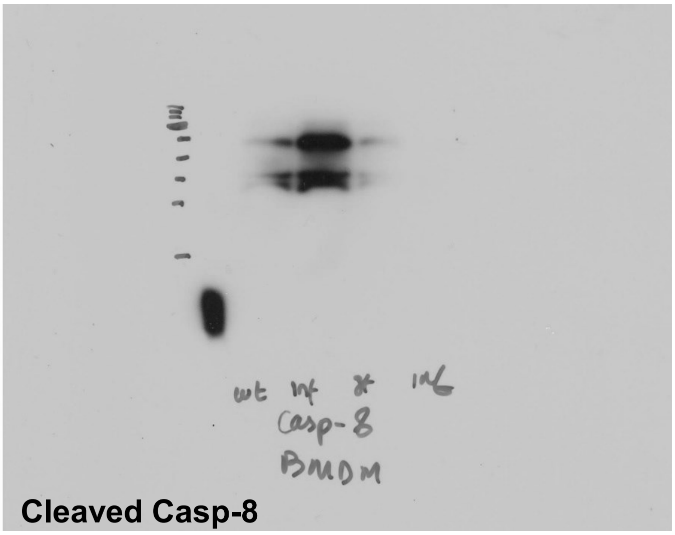

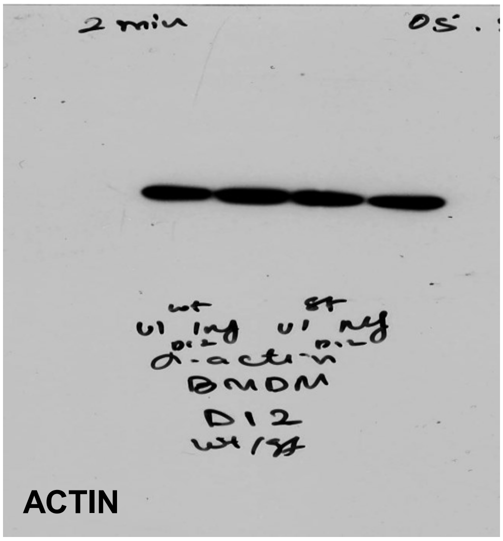

Related to Figure-3I: Uncropped western blots

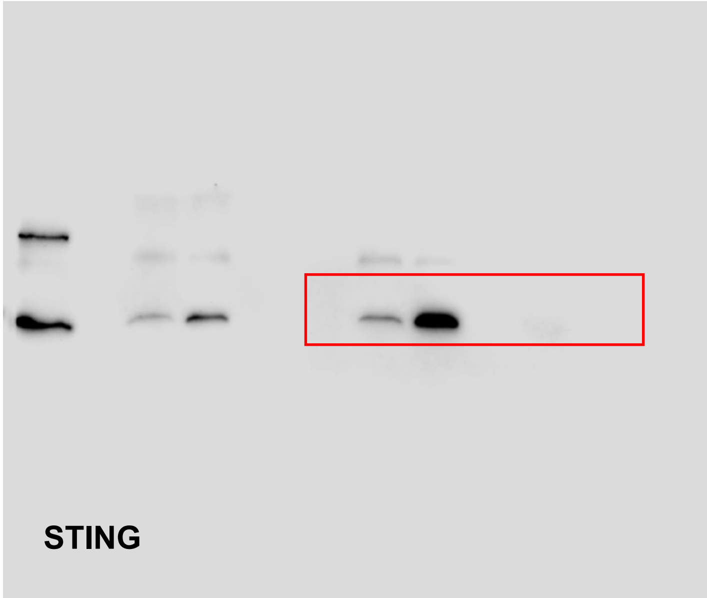

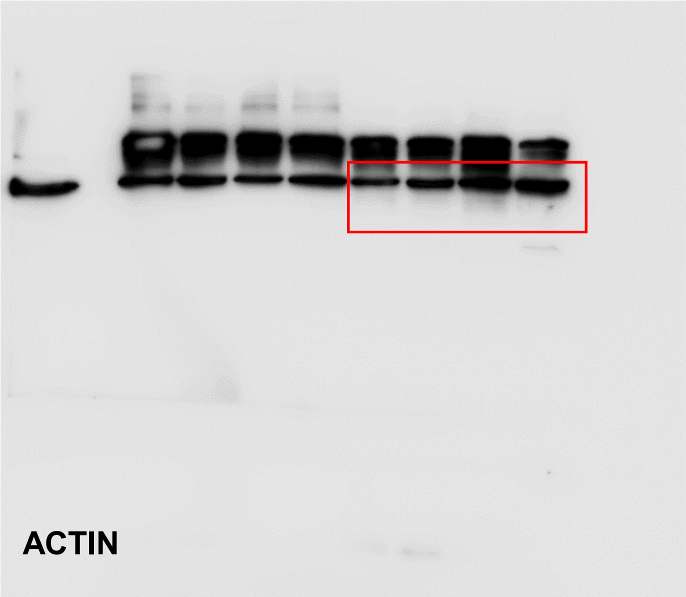

Related to Figure-4B: Uncropped western blots

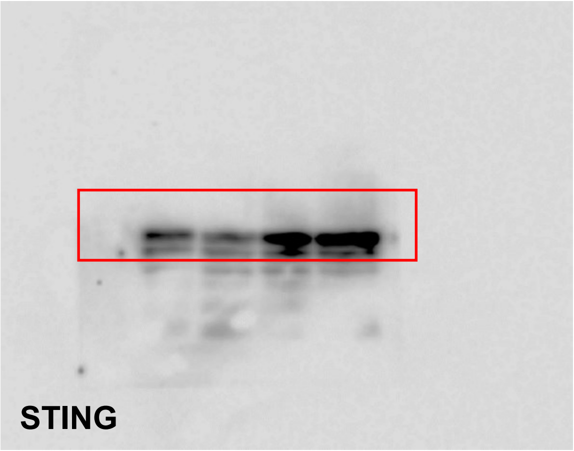

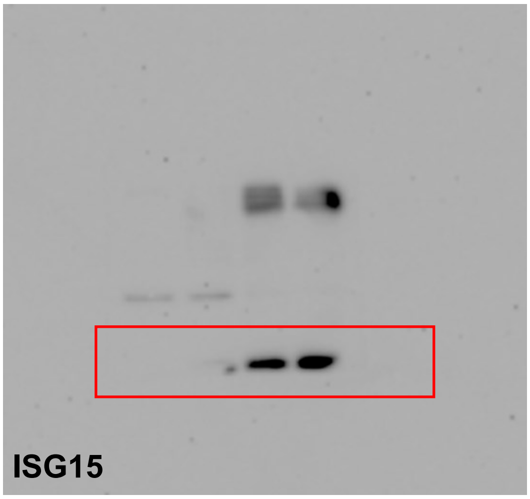

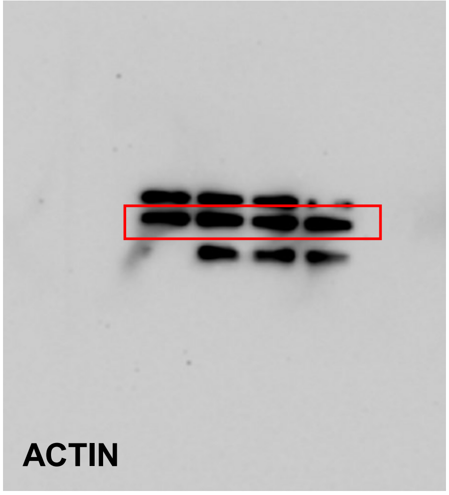

Related to Figure-4C: Uncropped western blots

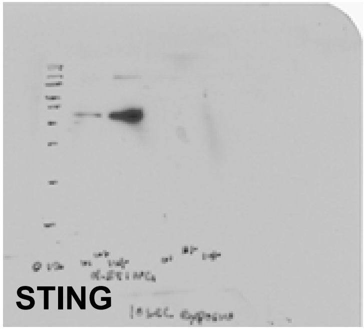

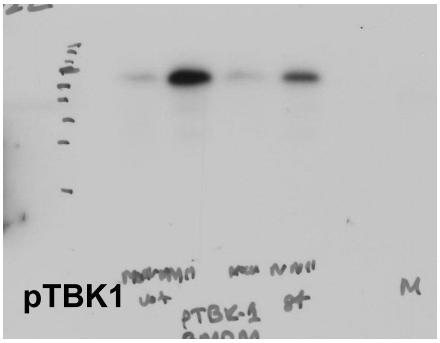

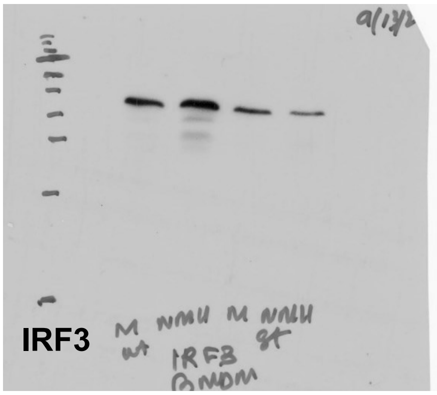

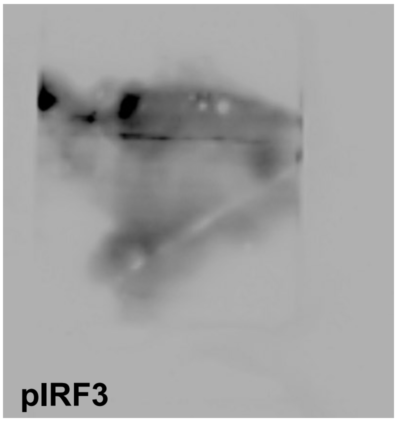

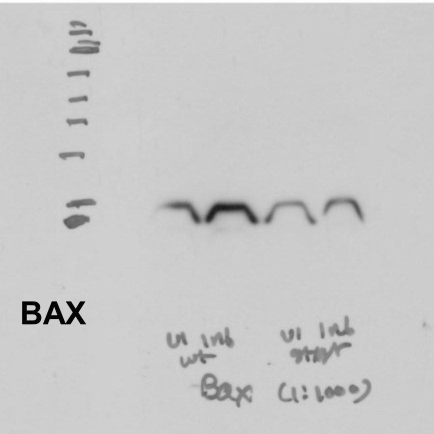

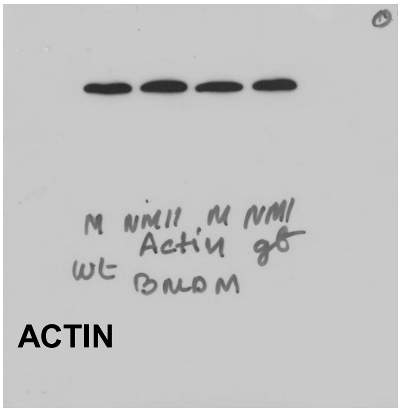

Related to Figure-4F: Uncropped western blots

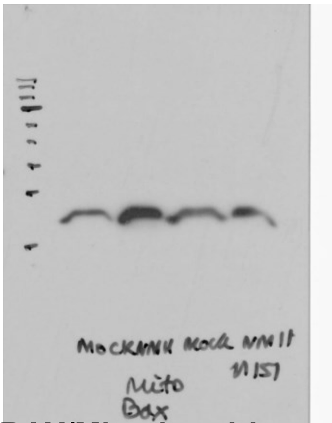

BAX/Mitochondria

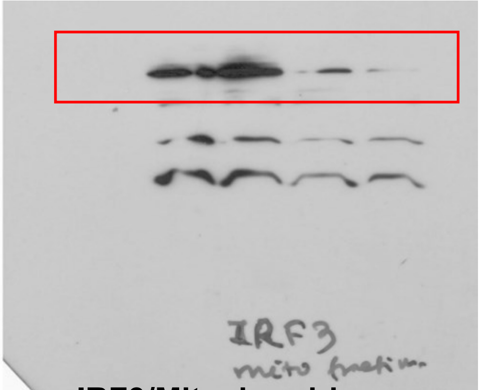

IRF3/Mitochondria

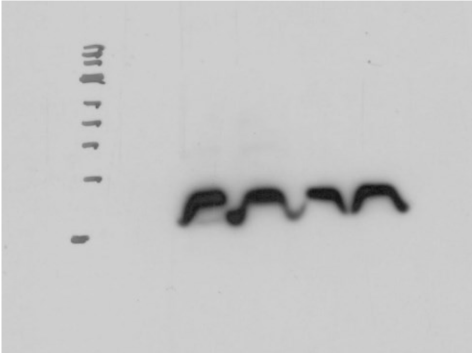

Cox-IV/Mitochondria

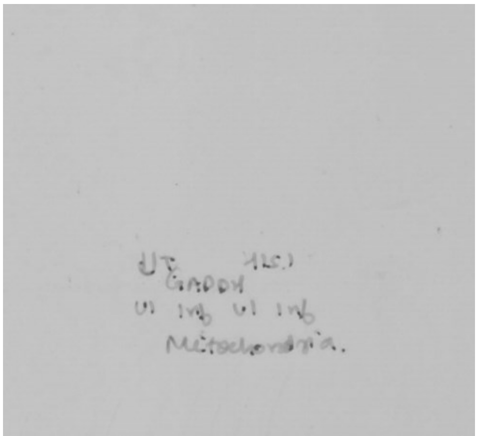

GAPDH/Mitochondria

Related to Figure-4H: Uncropped western blots

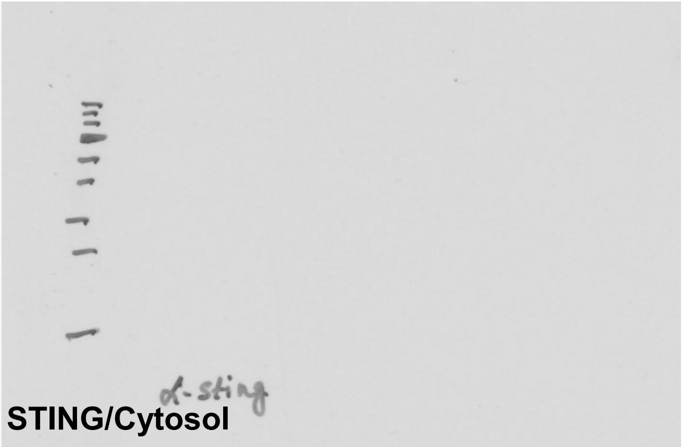

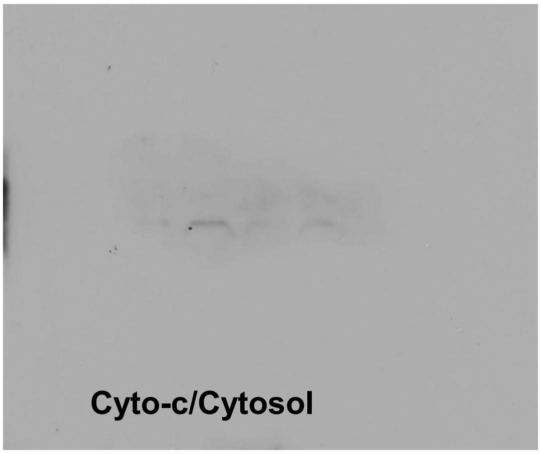

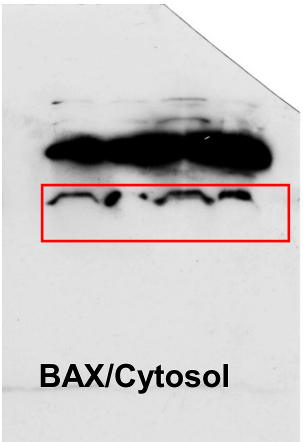

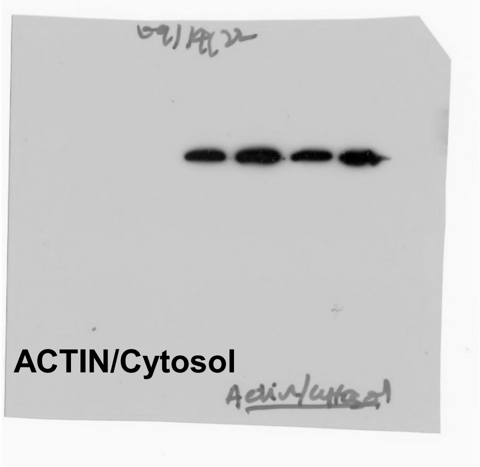

Related to Figure-4I: Uncropped western blots

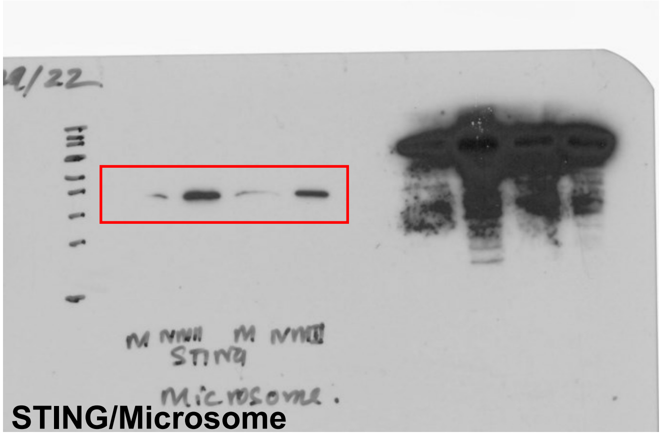

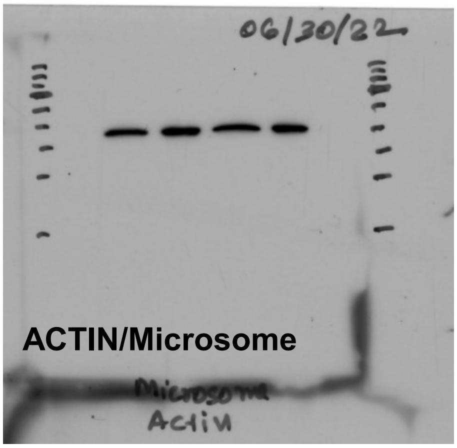

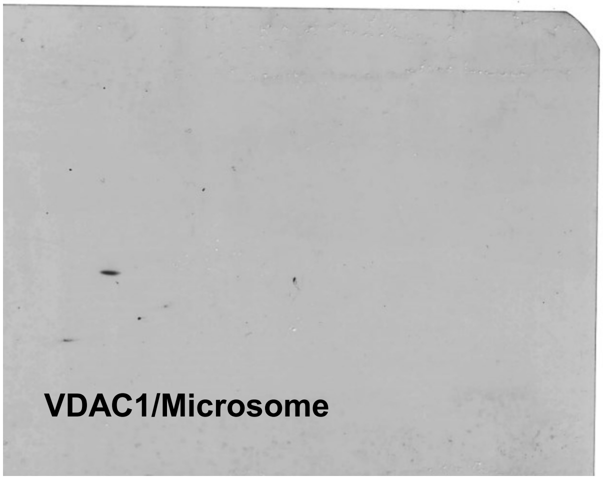

Related to Figure-4J: Uncropped western blots

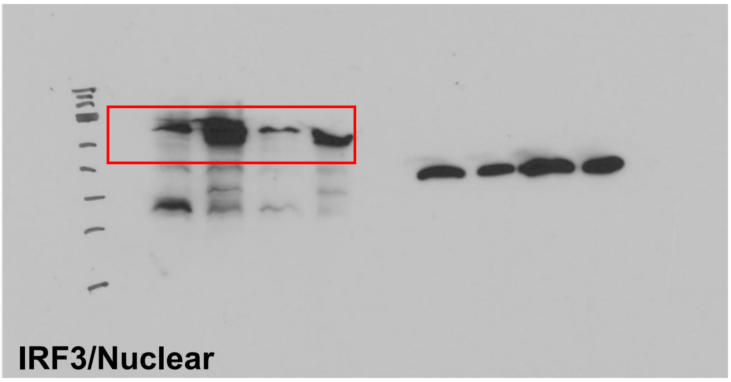

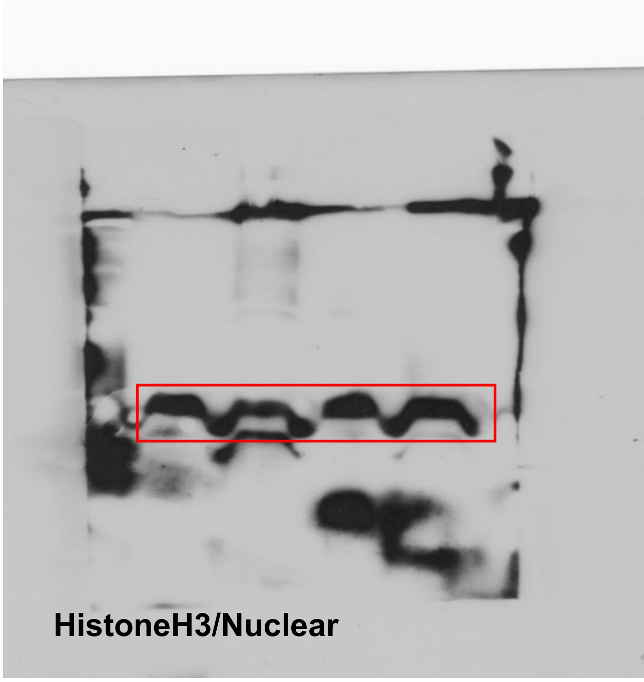

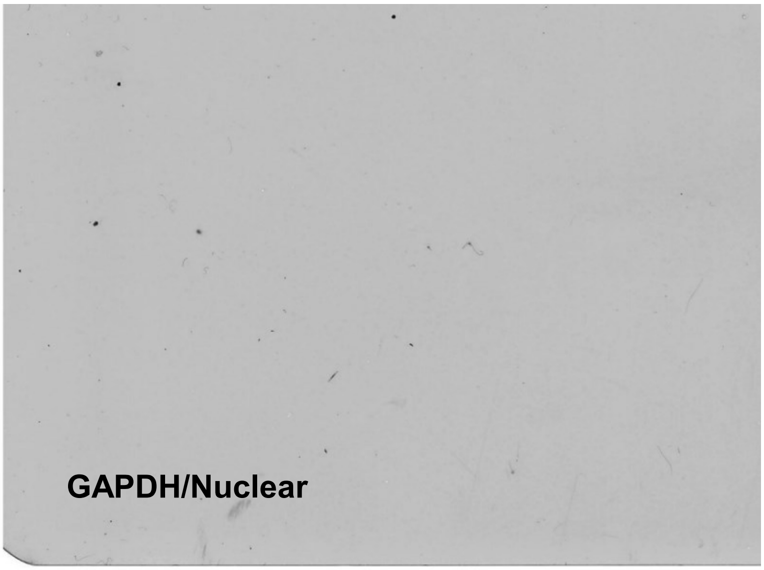

Related to Figure-4K: Uncropped western blots

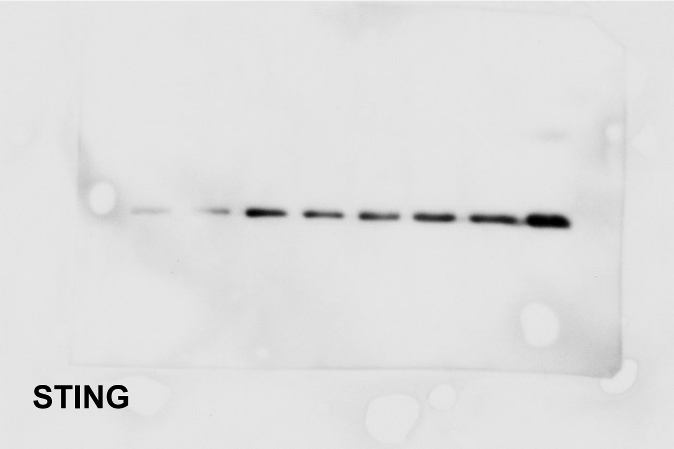

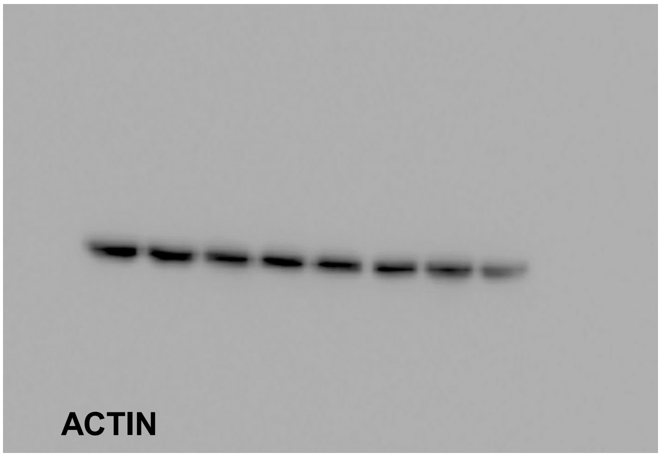

Related to Supplementary Figure-4A: Uncropped western blots

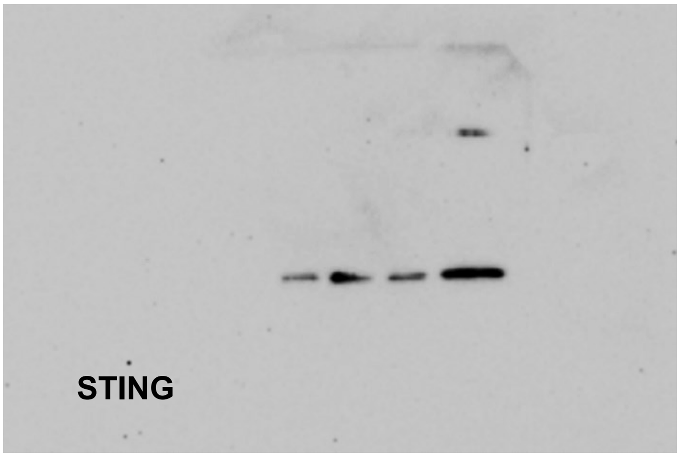

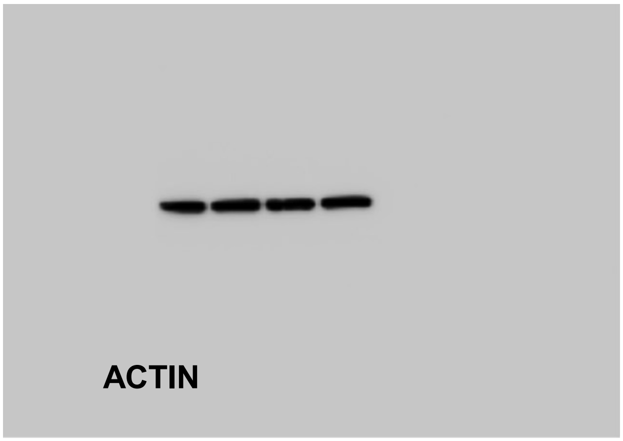

Related to Supplementary Figure-4B: Uncropped western blots

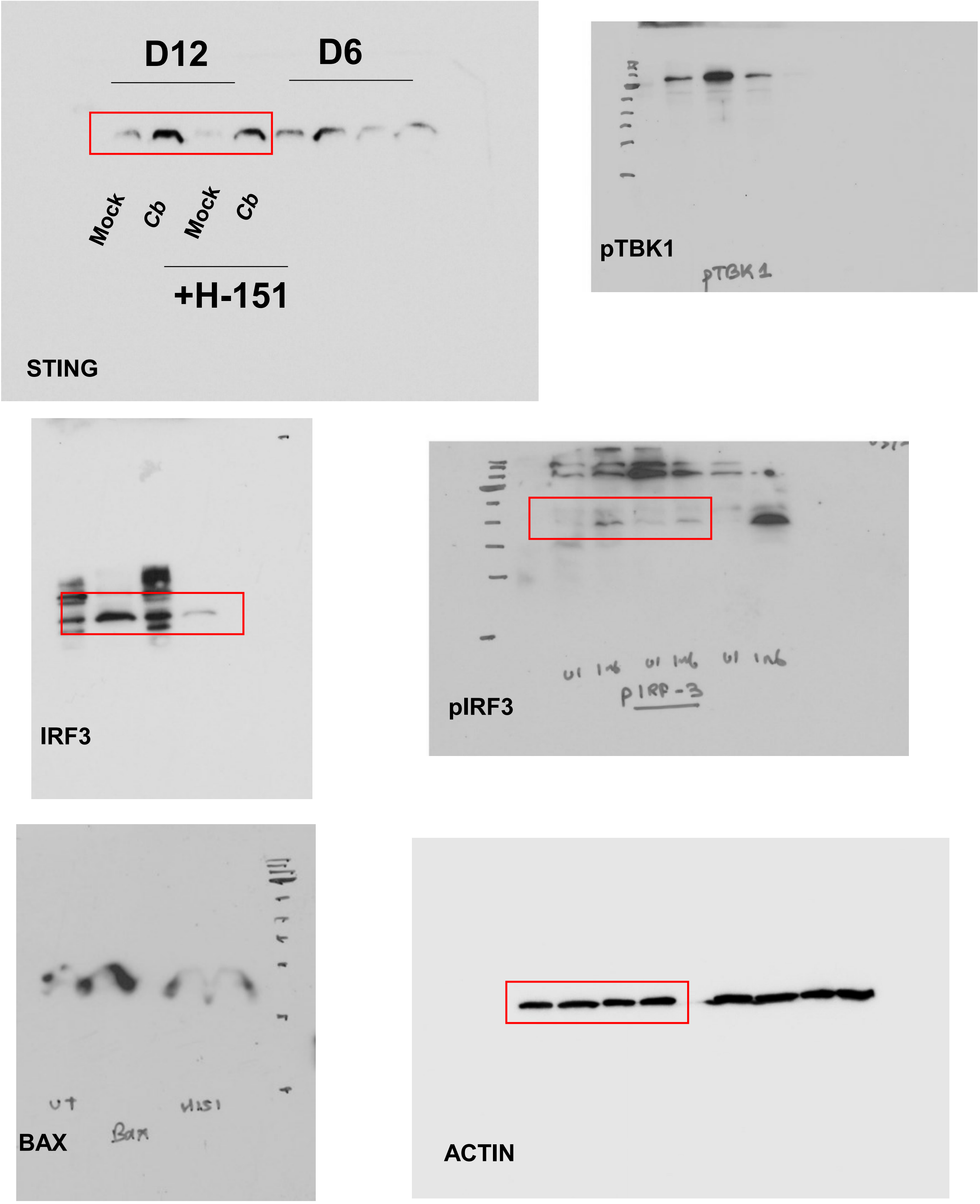

Related to Supplementary Figure-4D: Uncropped western blots

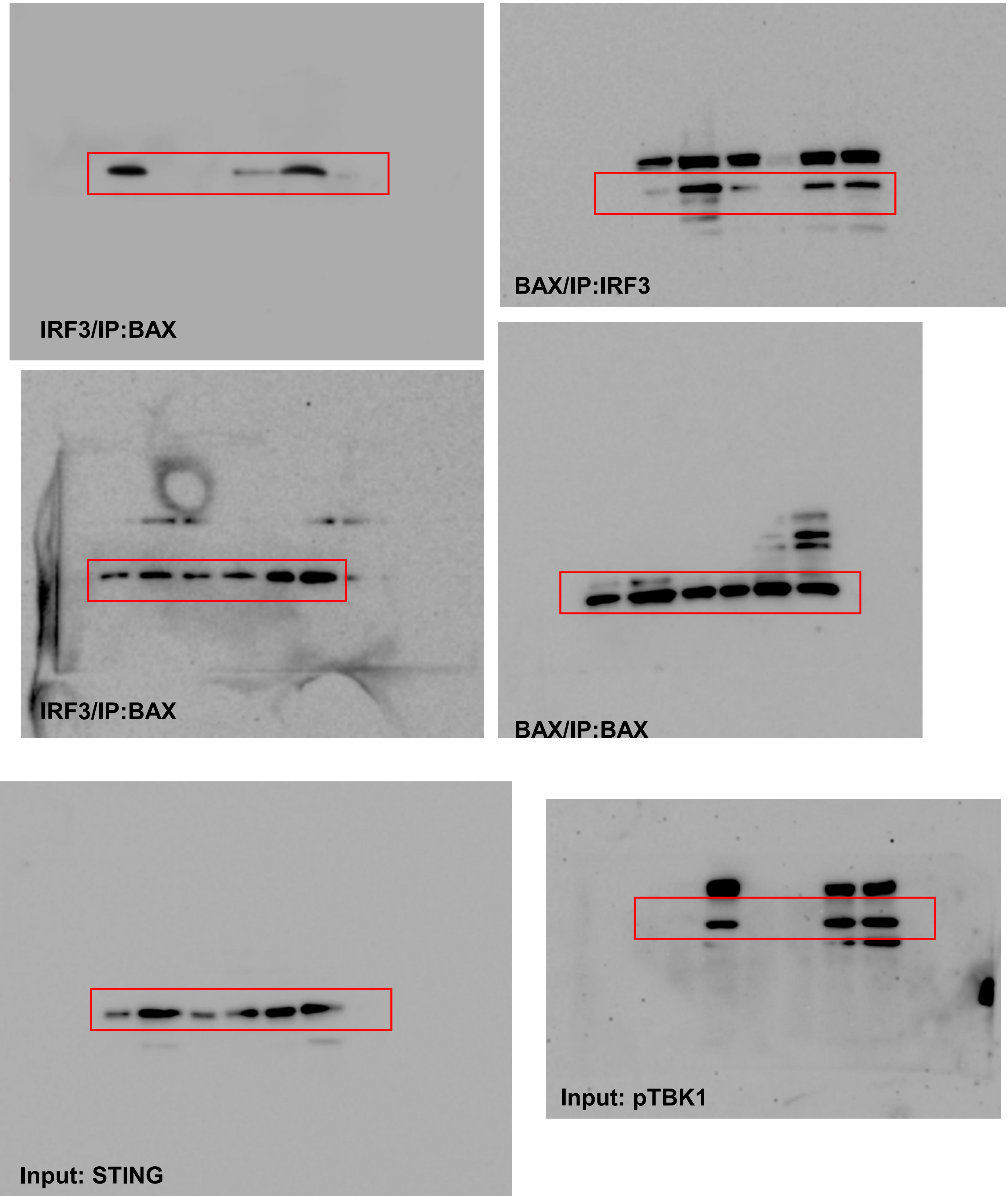

Related to Figure-5C: Uncropped western blots

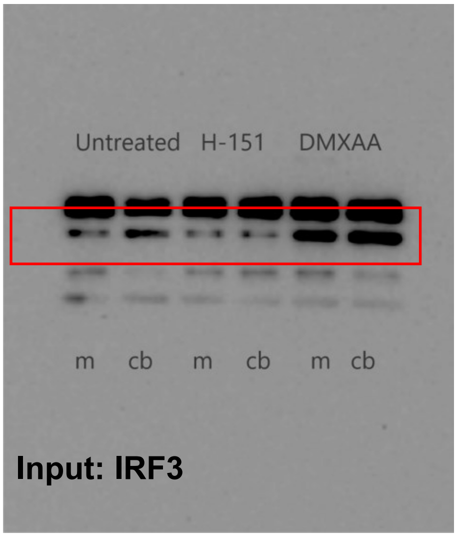

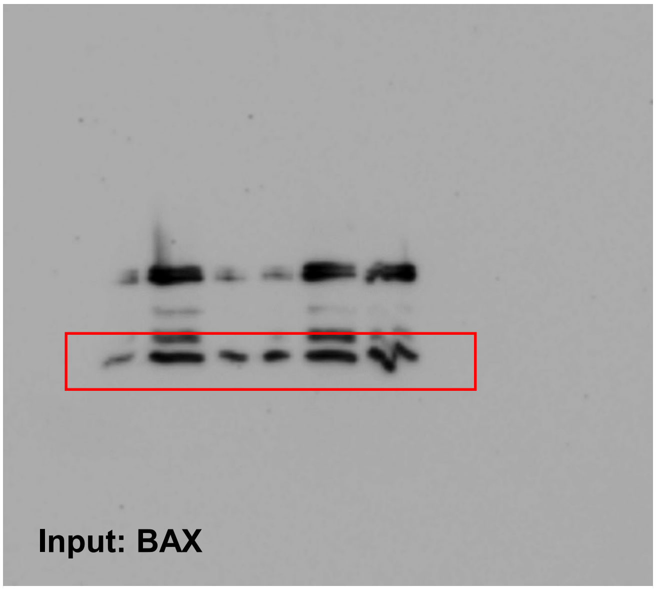

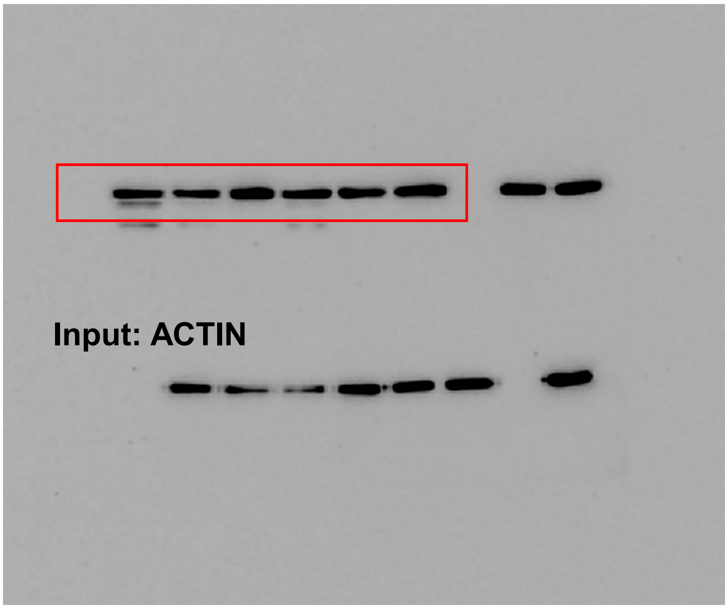

Related to Figure-5C: Uncropped western blots

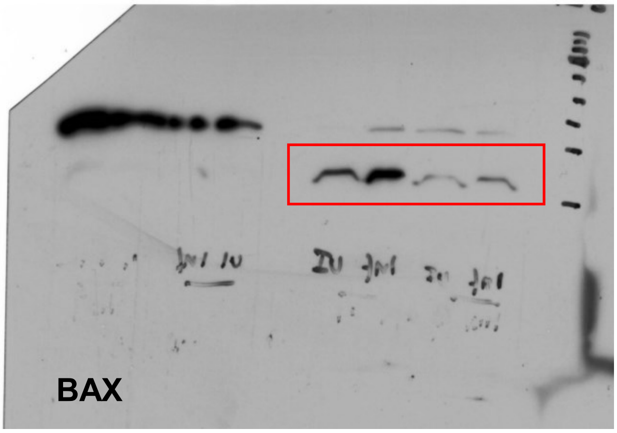

BAX

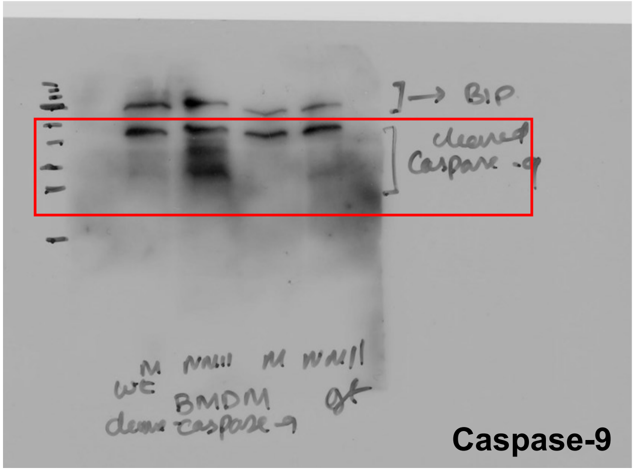

Caspase-9

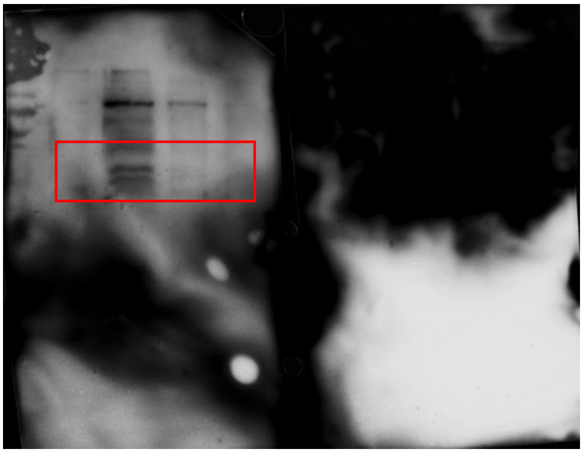

Cleaved-caspase-3

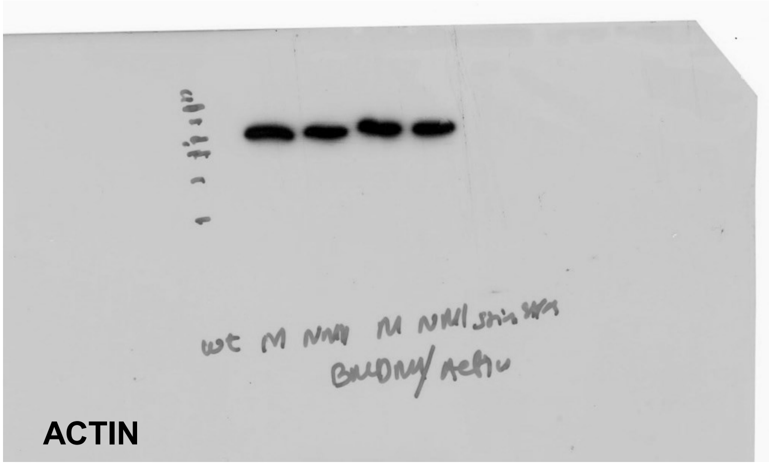

ACTIN

Related to Figure-6G: Uncropped western blots

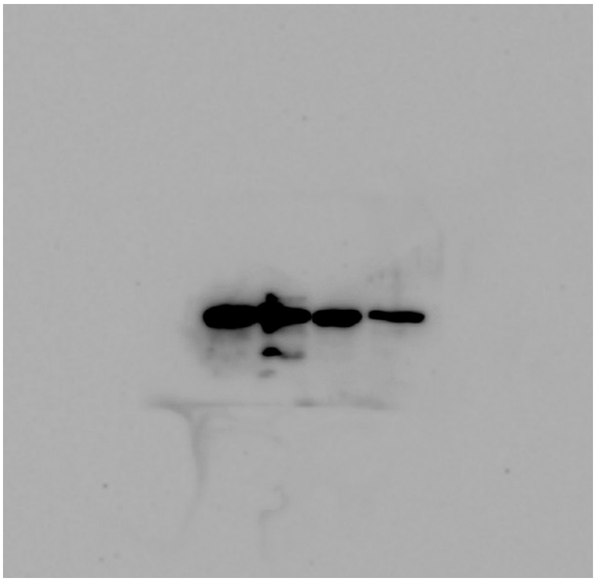

Caspase-9

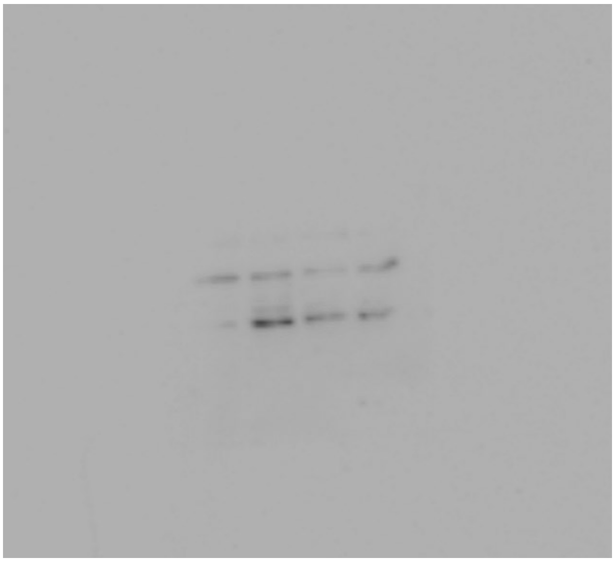

Caspase-8

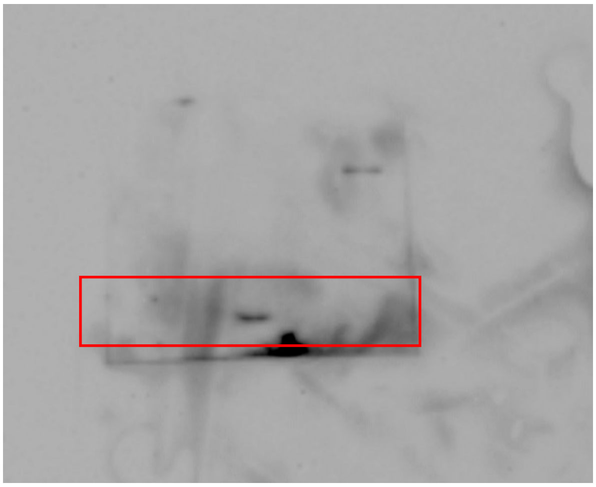

Cleaved Casp-3

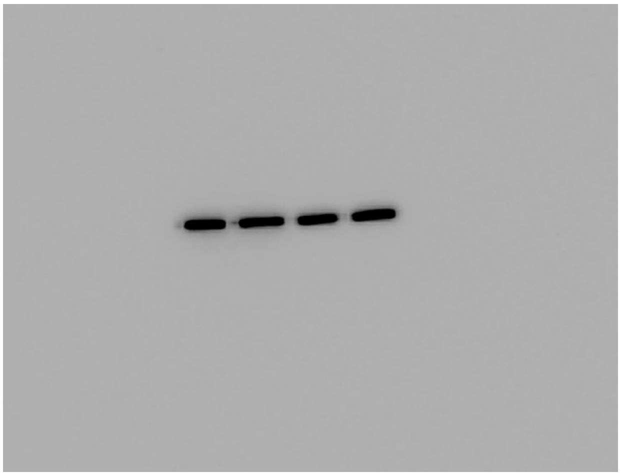

ACTIN

Related to supplementary Figure-6C: Uncropped western blots

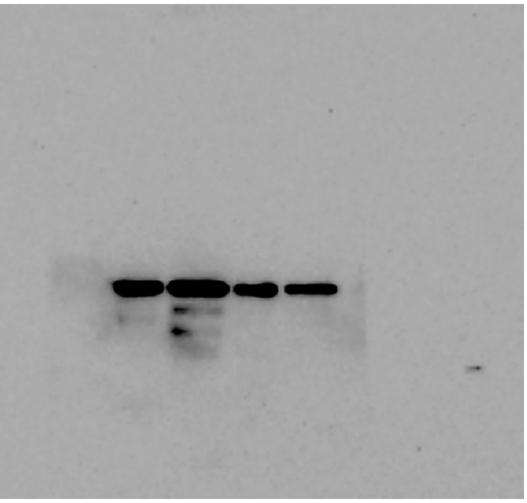

Casapse-9

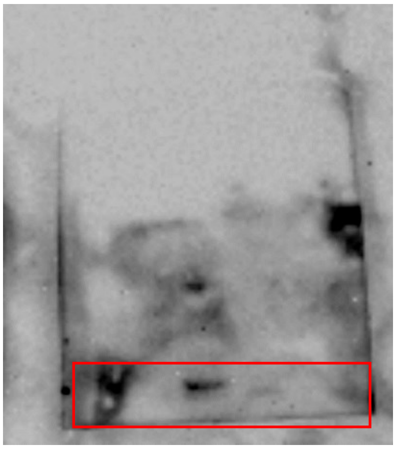

Cleaved Casp-3

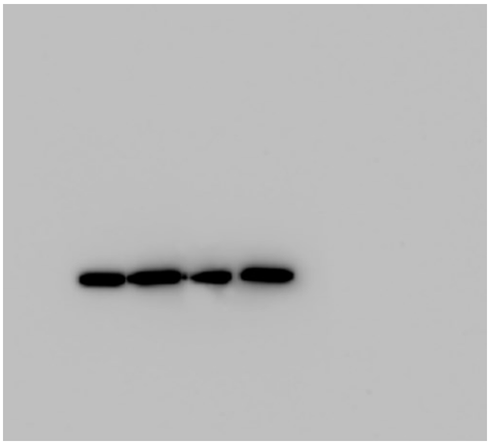

ACTIN

Related to supplementary Figure-6D: Uncropped western blots

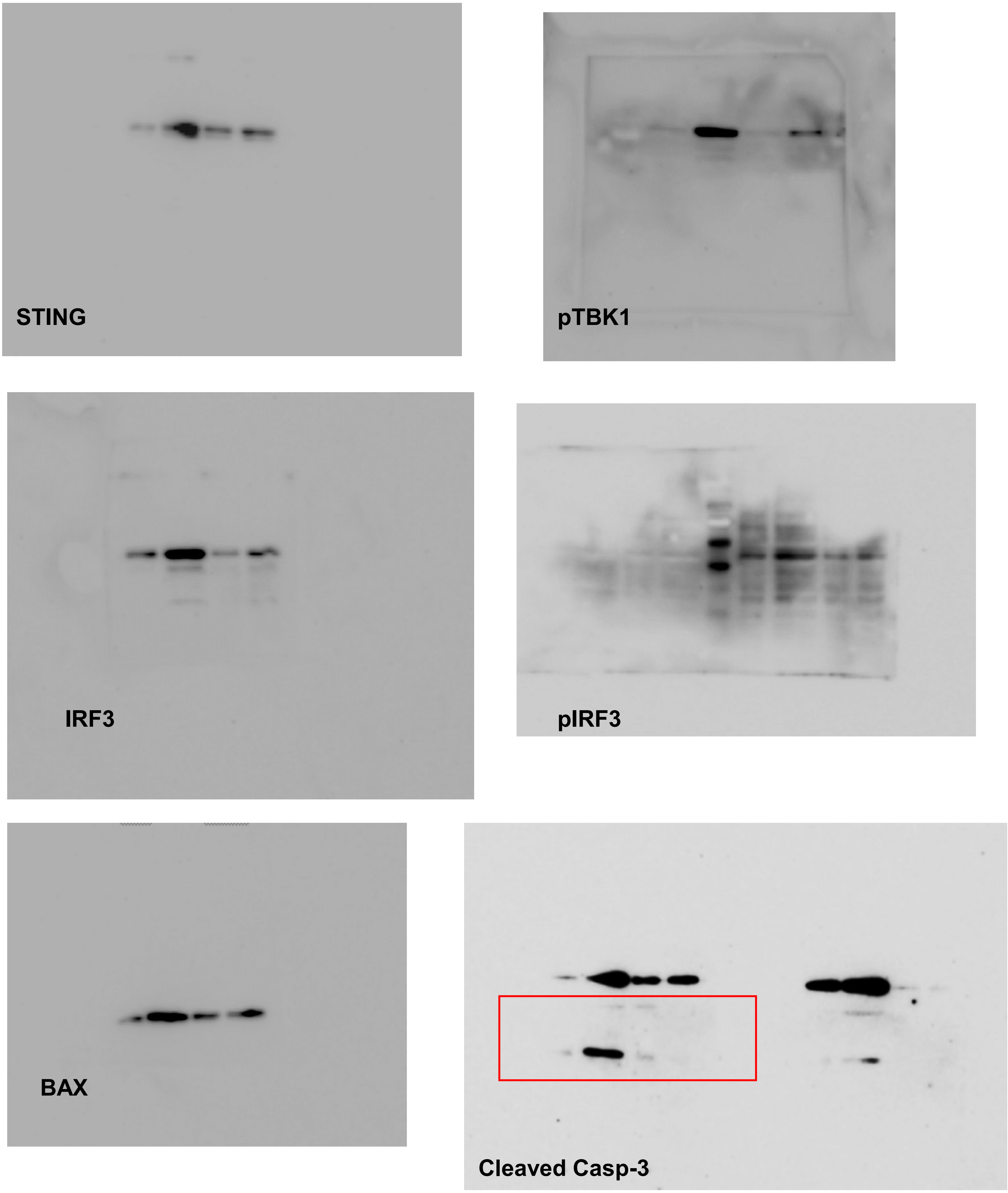

Related to Figure-8D: Uncropped western blots

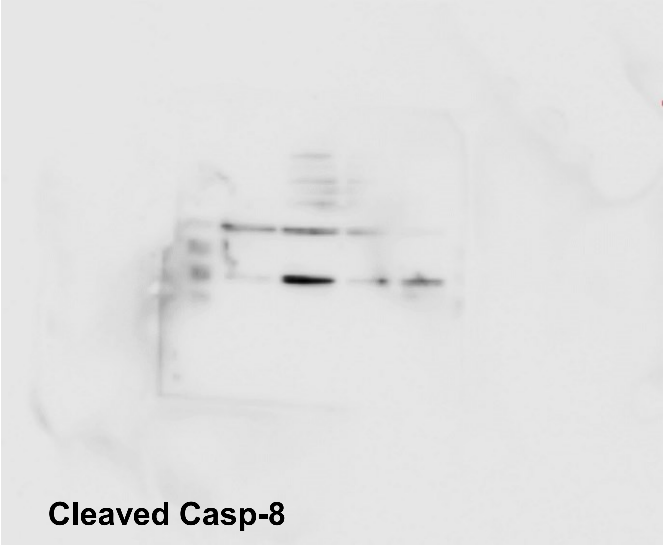

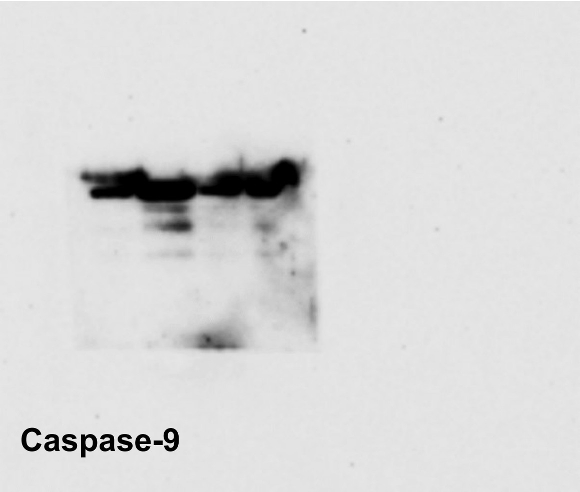

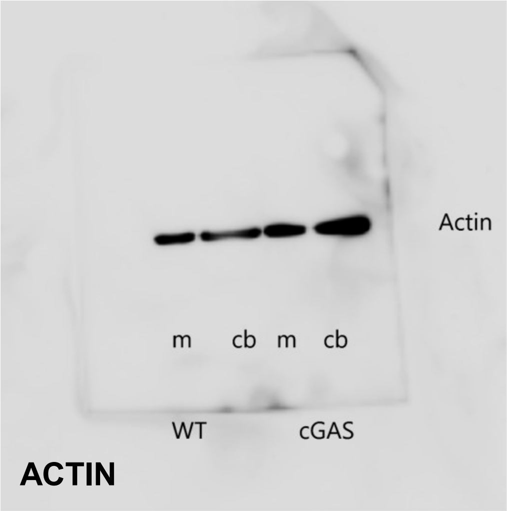

Related to Figure-8D: Uncropped western blots
